# Supplementary material for: Geographical variation in rates of surgical treatment for female stress urinary incontinence in England: a national cohort study
Source: BMJ Open. 2019 Aug 28;9(8):e029878. doi: 10.1136/bmjopen-2019-029878 (PMC6720135; doi:10.1136/bmjopen-2019-029878)
Supplement: Author's manuscript [file bmjopen-2019-029878.draft_revisions.pdf]

BMJ Open

BMJ Open is committed to open peer review. As part of this commitment we make the peer review history of every article we publish publicly available.

When an article is published we post the peer reviewers' comments and the authors' responses online. We also post the versions of the paper that were used during peer review. These are the versions that the peer review comments apply to.

The versions of the paper that follow are the versions that were submitted during the peer review process. They are not the versions of record or the final published versions. They should not be cited or distributed as the published version of this manuscript.

BMJ Open is an open access journal and the full, final, typeset and author-corrected version of record of the manuscript is available on our site with no access controls, subscription charges or pay-per-view fees (http://bmjopen.bmj.com).

If you have any questions on BMJ Open's open peer review process please email info.bmjopen@bmj.com

BMJ Open

GEOGRAPHICAL VARIATION IN RATES OF SURGICAL TREATMENT FOR FEMALE STRESS URINARY INCONTINENCE IN ENGLAND: A NATIONAL COHORT STUDY

Journal:	*BMJ Open*
Manuscript ID	bmjopen-2019-029878
Article Type:	Research
Date Submitted by the Author:	18-Feb-2019

Complete List of Authors:	Mamza, Jil; London School of Hygiene and Tropical Medicine Faculty of Public Health and Policy, Department of Health Services Research and Policy<br>Geary, Rebecca; London School of Hygiene and Tropical Medicine Faculty of Public Health and Policy, Department of Health Services Research and Policy; University College London, Department of Infection and Population Health<br>El-Hamamsy, Dina; University Hospitals of Leicester NHS Trust, Obstetrics and Gynaecology Department<br>Cromwell, David; London School of Hygiene and Tropical Medicine<br>Duckett, Jonathan; Medway NHS Foundation Trust<br>Monga, Ash ; University of Southampton<br>Toozs-Hobson, Philip; Birmingham Women's and Children's NHS Foundation Trust<br>Mahmood, Tahir; Forth Park Hospital, Obstetrics and Gynaecology<br>Wilson, Andrew; University of Leicester, Health Sciences<br>Tincello , Doug ; University of Leicester<br>van der Meulen, Jan; London School of Hygiene and Tropical Medicine<br>Gurol Urganci, Ipek; London School of Hygiene & Tropical Medicine, Health Services Research & Policy

Keywords:	Urogynaecology < GYNAECOLOGY, Geographical mapping < TROPICAL MEDICINE, Minimally invasive surgery < GYNAECOLOGY

SCHOLARONE™
Manuscripts

GEOGRAPHICAL VARIATION IN RATES OF SURGICAL TREATMENT FOR FEMALE STRESS URINARY INCONTINENCE IN ENGLAND: A NATIONAL COHORT STUDY

Jil B Mamza[1*], Rebecca S Geary[1*], Dina El-Hamamsy[2], David Cromwell[1], Jonathan Duckett[3], Ash Monga[4], Philip Toozs-Hobson[5], Tahir Mahmood[6], Andrew Wilson[7], Douglas G Tincello[7], Jan van der Meulen[1#], Ipek Gurol-Urganci[1#]

[1] Department of Health Services Research and Policy, London School of Hygiene & Tropical Medicine

[2] Obstetrics and Gynaecology Department, University Hospitals of Leicester

[3] Medway NHS Foundation Trust

[4] University Hospital Southampton NHS Foundation Trust

[5] Birmingham Women's NHS Foundation Trust

[6] NHS Fife, Victoria Hospital

[7] Department of Health Sciences, University of Leicester

[*] *Joint first authors have made an equal contribution to this study and manuscript*

[#] *Joint senior authors have made an equal contribution to this study and manuscript*

Corresponding Author

Ipek Gurol-Urganci

Department of Health Services Research and Policy
London School of Hygiene & Tropical Medicine
15-17 Tavistock Place, London WC1H 9SH, United Kingdom
Telephone: +44 (0)20 7927 2266

Email: ipek.gurol@lshtm.ac.uk

Word count: 2257 words

Running title: Variation in surgery for female stress urinary incontinence

For peer review only

ABSTRACT

Objective To examine geographic variation in use of surgery for female stress urinary incontinence (SUI), mainly mid-urethral mesh tape insertions, in the English National Health Service (NHS).

Design National cohort study

Setting NHS hospitals

Participants 27,997 women aged 20 years or older who had a first stress urinary incontinence surgery in an English NHS Hospital between April 2013 and March 2016 and a diagnosis of stress urinary incontinence at the same time as the procedure

Methods Multilevel Poisson regression was used to adjust for geographic differences in age, ethnicity, prevalence of long-term illness and socio-economic deprivation.

Primary outcome measure Rate of surgery for stress urinary incontinence per 100,000 women/year at two geographic levels: Clinical Commissioning Group (CCG, n=209) and Sustainability and Transformation Partnership (STP, n=44).

Results The rate of surgery for stress urinary incontinence was 40 procedures per 100,000 women/year. Risk-adjusted rates ranged from 20 to 106 procedures per 100,000 women/year across CCGs and 24 to 69 procedures per 100,000 women/year across the STP areas. These regional differences were only partially explained by demographic characteristics as adjustment reduced variance of surgery rates by 16% among the CCGs and 35% among the STPs.

Conclusions Substantial geographic variation exists in use of surgery for female stress urinary incontinence in the English NHS, suggesting that women in some areas are more likely to be treated compared to women with the same condition in other areas. The variation reflects differences in how national guidelines are being interpreted, in the context of the ongoing debate about the safety of SUI surgery.

Funding National Institute for Health Research (NIHR) Health Services and Delivery Research (HS&DR) Programme (14/70/162)

Keywords: Stress urinary incontinence, mid-urethral mesh tape, geographical variation.

For peer review only

Article Summary

Strengths and limitations of this study

- The data used for the study includes all surgical procedures performed within English NHS Hospitals, reducing the risk of selection bias
- Statistical modelling, utilising multi-level empirical Bayes methods was used to minimise potential estimation error problems when identifying potential outlier areas
- Unmeasured confounding factors and differences in coding practices may have contributed to variation in surgery rates
- This study did not account for surgical procedures performed in private hospitals. However, it is likely that at least 90% of all continence procedures in England are provided by the NHS, as the total annual spend on private health care in England is approximately 5% of the total annual spend on the NHS.

For peer review only

INTRODUCTION

Stress urinary incontinence (SUI), estimated to affect 15 to 20% of women in the UK(1), has a significant impact on women's quality of life,(2) affecting physical and social activities and confidence(3). Surgical treatments are recommended when conservative treatments are ineffective or not tolerated(4).

Mid-urethral mesh tapes were introduced in 1998 as a novel surgical treatment for female SUI(5). A sharp rise in the use of mesh tapes to treat SUI followed, due in part to the minimally invasive nature of the procedure, with a high of 11,365 procedures conducted in 2009. Over the same period, the previous standard treatment for female SUI, colposuspension (a major abdominal surgery) declined from more than 3,500 procedures per year to just 200(6). However, after the peak of more than 11,000 procedures in 2008-09, the number of mesh procedures for SUI has almost halved, falling to just 6227 by 2016-17(7, 8). The decline in the use of mesh tapes for SUI has most likely been in response to concerns about the safety of mesh(9-11) with some women experiencing pain, dyspareunia, persistent urinary incontinence, and exposure or erosion(12, 13). In 2018, the use of mesh tapes to treat SUI was suspended in the NHS in England, following an interim recommendation of the Independent Medicines and Medical Devices Safety Review(14, 15).

Previous studies highlighted that not all women with SUI have equitable access to appropriate incontinence care; access to continence surgery varies by age(16, 17) and ethnic and socio-economic backgrounds;(18) with evidence of variations in care for other vulnerable populations. In light of the current suspension of the use of mid-urethral mesh tapes, the most commonly used procedures to treat female SUI, evidence is needed regarding the utilisation, safety and effectiveness of mesh, and non-mesh, surgical continence procedures. Given the concerns about differences in access to surgical treatment for SUI, we assessed geographic variation in the rates of surgical treatment for female SUI in the English National Health Service (NHS) between 2013 and 2016 across two geographic levels, defined according to structures for commissioning services and coordinating service improvement.

For peer review only - http://bmjopen.bmj.com/site/about/guidelines.xhtml

METHODS

Study design, setting and definitions

This study used data from Hospital Episode Statistics (HES), a routinely collected, administrative dataset which contains records of all NHS hospital admissions in England. The cohort comprised women aged 20 years and older who had received surgical treatment for SUI between 1 April 2013 and 31 March 2016 and had an SUI diagnosis recorded at the time of the procedure. SUI surgery was defined using UK Office for Population Censuses and Surveys Classification (OPCS-4) codes (Table 1)(19). SUI diagnosis was defined using the International Classification of Diseases, ICD-10 code: N39.3 Stress urinary incontinence(20). Women may have had repeat procedures in the study period, however, only the first operation was counted in calculating the rate of surgery.

Measures

The outcome measure was rate of surgery for SUI per 100,000 women/year at two geographic levels: 209 Clinical Commissioning Group (CCG) and 44 Sustainability and Transformation Partnership (STP) areas. CCGs are statutory NHS bodies responsible for the planning and commissioning of health care services in a local area (average population size of about 104,000 adult females). CCG areas are grouped into 44 STP areas (average population size of about 493,000 adult females), which were set up to coordinate improvements in the delivery of NHS services(21). Reference denominator populations were derived by aggregating the 2011 Census population counts for women aged 20 and older in lower super output areas (LSOA) that are within the respective boundaries of the CCG and STP areas. There are 32,844 LSOAs (postcode-based geographic units) in England (average population approximately 1,700 people)(22).

Sociodemographic factors may explain variations in rates of surgery for SUI. We handled age as a patient-level characteristic grouped into five categories (20-39, 40-49, 50-59, 60-69 and 70+ years). Socio-economic status, ethnicity and limiting long-term illness were CCG-level

For peer review only - http://bmjopen.bmj.com/site/about/guidelines.xhtml

characteristics derived from 2011 Census data. For socio-economic status, we used the averages of the national ranking of the Index of Multiple Deprivation (IMD) (23) of LSOAs within each CCG, and grouped the CCG averages into national quintiles ranging from 1 (most deprived CCGs) to 5 (least deprived CCGs). For ethnicity, we used the percentage of the population reporting black or ethnic minority (BME) background, and for long-term illness the percentage who reported that their day-to-day activities were limited because of a health problem or disability which has lasted, or is expected to last, at least 12 months. For each CCG, we took the averages of these percentages for LSOAs and grouped these CCG averages into national quintiles ranging from 1 (CCGs with average percentages in the lowest quintile) to 5 (highest quintile).

Statistical analyses

We calculated the number and the unadjusted and adjusted rates per 100,000 women/year of SUI procedures overall, and according to patient and regional characteristics. Incidence rate ratios were used to represent associations between the procedure rate and regional characteristics. Multilevel Poisson regression models were used to produce empirical Bayes estimates of the unadjusted and adjusted incidence rates for each CCG and STP area. In addition, risk adjusted regression models were used to assess geographic variation in rates of surgery by year. The empirical Bayes estimator produces more precise results by “pulling” the estimates for small outlier regions towards the overall mean(24). For each area level (CCG/STP), we illustrated the amount of variation in adjusted surgery rates using maps and range plots with 99.8% credibility intervals. CCGs and STPs were marked as “outliers” where the national average rate of surgery was not within the 99.8% credibility interval of their rates. All statistical calculations were performed using Stata 14.

Patient involvement

This study was supported by a steering group which included lay members and patient representatives who provided input to the design of the study and interpretation of the results and contributed to the dissemination plan. The steering group met on a regular basis for the duration of the study.

For peer review only - http://bmjopen.bmj.com/site/about/guidelines.xhtml

RESULTS

Description of the cohort

Between April 2013 and March 2016, there were 33,708 inpatient episodes with a surgical procedure for SUI. 4,996 of these episodes did not satisfy the inclusion criteria, for example, because they did not have an SUI diagnosis recorded at the time of the procedure, outlined in Figure 1, and 715 recorded a subsequent operation in the study period (Figure 1). 27,997 procedures were included in the analyses, 90% of which were mid-urethral mesh tape insertions (Table 1). Restricting our analyses to these 27,997 first SUI procedures captured >97% of all 28,789 SUI procedures in the study period, and the distribution of procedure types did not vary between all and first procedures (Table 1). The national annual rate of surgery was 40 procedures per 100,000 women.

For peer review only

For peer review only - http://bmjopen.bmj.com/site/about/guidelines.xhtml

Table 1: OPCS-4 codes and counts of SUI procedures with relevant diagnosis (ICD-10) code N39.3

OPSC-4	Description	All operations* N (%)	First operations** N (%)
	**Mid-urethral tape insertions**		
M53.3	Introduction of tension-free vaginal tape	16665 (57.9)	16415 (58.6)
M53.6	Introduction of transobturator tape	8866 (30.8)	8722 (31.2)
	**Injection of urethral bulking agents**		
M56.3	Endoscopic injection of inert substance into outlet of female bladder	1628 (5.7)	1435 (5.1)
	**Other abdominal / vaginal operations**		
M51.1	Abdominoperineal suspension of urethra	32 (0.1)	29 (0.1)
M51.2	Endoscopic suspension of neck of bladder	6 (<0.1)	6 (<0.1)
M51.8	Other specified combined abdominal and vaginal operations to support outlet of female bladder	15 (0.1)	13 (<0.1)
M51.9	Unspecified combined abdominal and vaginal operations to support outlet of female bladder	2 (<0.1)	2 (<0.1)
M52.1	Suprapubic sling operation	355 (1.2)	328 (1.2)
M52.2	Retropubic suspension of neck of bladder	78 (0.3)	76 (0.3)
M52.3	Colposuspension of neck of bladder	587 (2.0)	533 (1.9)
M52.8	Other specified abdominal operations to support outlet of female bladder	20 (0.1)	15 (0.1)
M52.9	Unspecified abdominal operations to support outlet of female bladder	3 (<0.1)	2 (<0.1)
M53.1	Vaginal buttressing of urethra	130 (0.5)	126 (0.5)
M53.8	Other specified vaginal operations to support outlet of female bladder	302 (1.0)	216 (0.8)
M53.9	Unspecified vaginal operations to support outlet of female bladder	5 (<0.1)	4 (<0.1)
M55.2	Implantation of artificial urinary sphincter into outlet of female bladder	18 (0.1)	11 (<0.1)
M55.6	Insertion of retropubic device for female stress urinary incontinence NEC	56 (0.2)	52 (0.2)
M55.8	Other specified other open operations on outlet of female bladder	14 (<0.1)	8 (<0.1)
M55.9	Unspecified other open operations on outlet of female bladder	0 (0)	0 (0)
M58.8	Other specified other operations on outlet of female bladder	7 (<0.1)	4 (<0.1)
M58.9	Unspecified other operations on outlet of female bladder	0 (0)	0 (0)
	**Total**	28789	27997

Abbreviations: SUI (stress urinary incontinence); ICD-10 (International Classification of Diseases, version 10); OPCS (Office for Population Censuses and Surveys Classification); N39.3 (Stress urinary incontinence ICD-10 code). *For 77 episodes of care out of 28712 eligible episodes, two procedures were recorded and both are included in the overall count. ** For episodes of care where two procedures were recorded, only the more invasive or specified procedure is counted as first operation.

For peer review only

Geographic variations in surgery

Figures 2a and 3a show the variation in the unadjusted empirical Bayes estimates of the procedure rates for SUI across the CCGs and STPs, with figures 2b and 3b illustrating the rates adjusted for patients' age and the CCG-level characteristics: socio-economic status, percentage of the population reporting BME background, and percentage with a long-term illness.

The adjusted SUI procedure rates for CCGs ranged from 20 to 106 procedures compared with unadjusted rates of 11 to 120 procedures per 100,000 women/year (Figure 2). Ninety-nine CCGs (47%) were marked as "outliers" (where the national average was not within the 99.8% credibility interval of their rate). These comprised 43 CCGs (20.6%) with rates below the national average and 56 CCGs (26.8%) with rates above the national average. Risk adjustment reduced the number of CCGs marked as "outliers" from 99 (47.4%) to 75 (36%). The standard deviation (SD) of the CCG-level variation in adjusted rates (SD 0.27, 95%CI: 0.24-0.30) was 16% lower than the SD of the unadjusted rates (SD 0.32, 95%CI: 0.29-0.36).

The adjusted SUI procedure rates for STPs ranged from 24 to 69 procedures compared with unadjusted rates of 20 to 77 per 100 000 women/year (Figure 3). Risk adjustment reduced the number of STPs identified as outliers from 23 (52%) to 22 (50%). The amount of variation observed declined by 35% after risk adjustment: the SD of the STP-level variation for unadjusted and adjusted model were 0.23 (95%CI: 0.17-0.31) and 0.15 (95%CI: 0.11-0.22) respectively.

Annual SUI procedure rates declined over the study period from 52 per 100,000 women in 2013 to 36 per 100,000 women in 2015. However, there was no evidence that CCG- or STP-level variation changed over time. In separate (adjusted) regression models run by year, the SD of CCG-level variation was 0.26 (95%CI: 0.23-0.30) in 2013; 0.27 (95%CI: 0.23-0.31) in 2014 and 0.29 (95%CI: 0.25-0.34) in 2015. The SD of STP-level variation (adjusted model) was 0.13 (95%CI: 0.08-0.20) in 2013, 0.17 (95%CI: 0.11-0.25) in 2014 and 0.18 (95%CI: 0.12-0.26) in 2015.

For peer review only

Association of patient and regional characteristics with surgery rates

Table 2 shows the rates of surgery by regional characteristics. Rates were lowest for the 20-39 year age group (16 per 100,000 women/year), and highest for 40-49 year age group (84 per 100,000 women/year), declining with age beyond 50 years. Compared to the rate among women aged 40-49 years, the surgery rate for women aged 50-59 years was 20% lower (IRR 0.80, 95% CI 0.78-0.83), for women aged 60 to 69 years was 46% lower (IRR 0.54, 95% CI 0.52-0.56), and for women aged 70+ years was 69% lower (IRR 0.31, 95% CI 0.30-0.33).

Rates of surgery were lower for areas with higher proportions of BME populations (highest vs lowest quintile IRR 0.63, 95% CI 0.49-0.81). There were no differences in surgery rates according to the proportion of people with long-term limiting illness or socioeconomic deprivation at the CCG-level.

For peer review only - http://bmjopen.bmj.com/site/about/guidelines.xhtml

Table 2: Regional characteristics and their association with SUI procedure rates

Regional factor	Scale of factor (1 unit)	Procedures, n (%)	Crude rate per 100,000 women/year	Procedure rate ratio (95%CI)	*P* value*
**Age categories**					
20-39	Age group in years	3253 (11.6)	15.9	0.18 (0.17-0.19)	
40-49		9761 (34.9)	84.4	Reference	<0.001
50-59		7496 (26.8)	67.5	0.80 (0.78-0.83)	
60-69		4352 (15.5)	46.2	0.54 (0.52-0.56)	
≥70		3135 (11.2)	26.8	0.31 (0.30-0.33)	
**Socio-economic status**					
Most deprived	Quintile category of IMD ranking	5838 (20.9)	43.0	Reference	0.84
More deprived		6315 (22.6)	47.5	1.08 (0.93-1.25)	
Average		6371 (22.8)	47.9	1.05 (0.89-1.25)	
Less deprived		5001 (17.9)	39.9	1.02 (0.85-1.21)	
Least deprived		4472 (15.1)	36.3	1.05 (0.85-1.29)	
**Black and minority ethnic (BME) population**					
1: CCGs with lowest proportion	Ranked category of proportion of BME population	5579 (19.9)	48.8	Reference	0.001
2		6867 (24.5)	49.8	1.02 (0.89-1.17)	
3		6326 (22.6)	45.7	1.00 (0.86-1.17)	
4		5725 (20.4)	41.5	0.89 (0.75-1.06)	
5: CCGs with highest proportion		3500 (12.5)	27.2	0.63 (0.49-0.81)	
**Limiting long-term illness**					
1: CCGs with lowest proportion	Ranked category of proportion of people with limiting illness	4433 (15.8)	32.8	Reference	0.46
2		6328 (22.6)	44.4	1.16 (0.99-1.36)	
3		4882 (17.4)	43.7	1.11 (0.91-1.34)	
4		6896 (24.6)	46.1	1.12 (0.91-1.39)	
5: CCGs with highest proportion		5458 (19.5)	48.9	1.16 (0.91-1.49)	

Random effects estimates	Standard deviation† (95%CI)	Standard deviation‡ (95%CI)
STP-level variation (level 2)	0.23 (0.17-0.31)	0.15 (0.11-0.22)
CCG-level variation (level 1)	0.32 (0.29-0.36)	0.27 (0.24-0.30)

Table 2 describes the distribution of regional characteristics and the association between these factors and SUI procedure rates from the multilevel random-intercept Poisson regression model. Abbreviations: CI (Confidence interval); IMD (Index of multiple deprivation); CCG (Clinical Commissioning Group); STP (Sustainability and Transformation Plan); NHS (National Health Service); †Unadjusted estimates; ‡Adjusted for all regional factors including ethnicity. *P value obtained from likelihood ratio test.

For peer review only

DISCUSSION

Main findings

More than 30,000 women were admitted to NHS hospitals in England for an SUI-related surgical treatment between April 2013 and March 2016. The rate of surgery for SUI was 40 procedures per 100,000 women/year. Crude regional rates varied by a factor of eleven among the 209 CCGs from 11 to 120, and a factor of four among 44 STPs from 20 to 77 procedures per 100,000 women/year. These differences were only slightly reduced when the women's age and regional characteristics were taken into account. The overall rate of SUI surgery dropped by a third over the three-year study period whilst the extent of geographic variation remained stable.

Interpretation

This study, carried out in the English NHS, is the first national study to explore geographic variation in rates of surgical treatment for SUI. Evidence to date regarding geographic variation in benign gynaecological surgery across England focused primarily on surgery for menorrhagia(25, 26) suggesting substantial variation despite the existence of national guidelines.

We found that women's age and regional ethnicity distributions were associated with surgery rates. This may reflect differences in incontinence-related health beliefs, preferences and care seeking behaviour for older women(27) and women from various ethnic backgrounds(18). Studies suggest that only around half of older people seek help for their incontinence symptoms, commonly due to the belief that it is a normal part of ageing.(28, 29) In England, studies concluded that help-seeking behaviour was hindered for South-Asian women as they felt embarrassed to discuss sensitive problems, particularly with a male health professional.(30, 31) Other studies in the Netherlands,(32) Sweden(33) and the USA(34) also found notable differences in preferences across women from different age groups and ethnic backgrounds.

For peer review only - http://bmjopen.bmj.com/site/about/guidelines.xhtml

We found that older women were less likely to have received surgical treatment for their SUI. This agrees with findings for other aspects of continence care. A national audit for continence care in the UK(17) found that deficiencies in the organisation of care and the management of urinary incontinence were more pronounced for older people.(35) For example, in acute and primary care settings, older people were less likely to have a continence history or focused examination done. In secondary care, whilst it has been shown that surgical treatments are safe and effective in older women,(36) these procedures were used less frequently than in younger patients(16, 17).

In their work on clinical practice variation, Wennberg and colleagues emphasise three factors as possible sources of variation: clinical uncertainty about the appropriateness of care, regional differences in patients' preferences for particular treatments , and differences in the capacity or supply of services(37). In the context of SUI surgery, a part of the observed variation will reflect the ongoing debate and concerns about the safety of mid-urethral mesh tape procedures for women with SUI, which in 2018 led to a 'pause' in the use of mesh for the treatment of stress urinary incontinence(9-11, 14, 15). It is important to note that adjustment for factors that are likely to affect patients' preferences had little impact on the geographic variation we observed for SUI surgery. However, patients' preferences will also be strongly guided by the advice received from their clinicians. The geographic areas used in this study (CCGs and STPs) are defined by NHS bodies that commission local hospital services which suggests that differences in capacity of the local healthcare system may have contributed to the observed variation.

For peer review only

The "correct" rate of SUI surgery is difficult to determine, especially given the ongoing concerns about the safety of mesh tapes. However, with the observed level of geographic variation, it is likely that women in some areas were more likely to be treated compared to women with the same condition in other areas. Informed patient choice, shared decision making and improved communication of the risks and benefits of both mesh and non-mesh procedures(38) is often proposed as a possible solution(37). NICE, the organisation that develops clinical guidelines for the English NHS, recommends a multidisciplinary team review prior to offering invasive therapy for SUI symptoms(4). In light of recent reviews, and the current suspension of mesh tape

For peer review only - http://bmjopen.bmj.com/site/about/guidelines.xhtml

insertions, NICE's latest draft guidance (published October 2018) also states that non-surgical options for SUI must be offered before any surgical treatment(39). A better understanding of relevant, long-term clinical outcomes is also needed(40, 41). With the current level of uncertainty about the safety and outcomes of mid-urethral mesh tape insertions, it is likely that the geographic variation we observed will continue.

In England, discussions are ongoing about setting up a national prospective registry of mid-urethral mesh tape insertions to monitor reoperations and removals as outcomes. However, there is also a clear need to capture a wider range of clinical outcomes that are directly relevant to women, including recurrent or persistent urinary incontinence, pain, and sexual dysfunction. These types of outcomes can only be collected if women themselves are actively involved in the process.

Strengths and limitations

The data used comprised information on all surgical procedures performed within the English NHS, thereby reducing the risk of selection bias. Our statistical modelling, utilising multi-level empirical Bayes methods allowed for minimising potential estimation error problems and taking account of area size variations for estimation of credibility intervals(24). This approach provided a powerful and statistically robust basis for identifying potential outlier areas.

Our analyses were subject to limitations inherent to observational studies. Firstly, unmeasured confounding factors may have contributed to variation in surgery rates. We were unable to account for potential regional variation in the average severity of the SUI problems. Secondly, whilst the overall quality of clinical information in HES has been found to be sufficiently high for research and audit purposes, inaccuracies in coding practices could have introduced some variation between geographic areas. Finally, we were unable to account for procedures done in the private sector. Although precise figures are lacking, it is likely that at least 90% of all incontinence procedures carried out in England are provided by the NHS, given that the total annual spending on private health care in England is about 5% of the total annual spending on

For peer review only - http://bmjopen.bmj.com/site/about/guidelines.xhtml

the NHS(42). While our study findings must be interpreted with caution in light of the above limitations, these are very unlikely to explain the large regional differences observed.

CONCLUSION

We found substantial variation in the rates of surgical treatment for female SUI between geographic regions across England. Adjusting for the women's age group and regional characteristics reduced variation only slightly. It is likely that the observed variation is in part linked to the ongoing debate about the safety of mid-urethral mesh tapes leading to differences in professional opinion about the appropriateness of surgical treatment for female SUI. This can only be informed by large-scale national studies monitoring long-term outcomes relevant to patients.

For peer review only

Acknowledgements

We would like to thank Lynn Copley and Natalie Eugene at the Clinical Effectiveness Unit of the Royal College of Surgeons of England and London School of Hygiene and Tropical Medicine for extracting the required data.

Disclosure of Interests

All authors received grant funding from the National Institute for Health Research (NIHR) Health Services and Delivery Research (HS&DR) Programme (14/70/162) during the conduct of the study. AM reports personal fees from: Astellas, Pfizer, Contura and Atlantic Medical; and PTH reports personal fees from: Boston Scientifc and Ethicon, and grants and personal fees from SEP Pharma outside the submitted work.

Contribution to Authorship

The study was conceived and designed by all authors (JBM, RSG, DEH, DC, JD, AM, PTH, TM, AW, DGT, JvdM, IGU). JBM and IGU organised the datasets and performed the statistical analysis, JBM wrote the first draft of the manuscript; RSG, IGU, JBM and JvdM wrote the final manuscript, with input from DEH, DC, JD, AM, PTH, TM, AW, DGT. All authors (JBM, RSG, DEH, DC, JD, AM, PTH, TM, AW, DGT, JvdM, IGU) contributed to the interpretation of results and approved the final text. Joint senior authors (IGU and JvdM) and joint first authors (JBM and RSG) made an equal contribution to this study and manuscript.

Patient Consent and Ethics Approval

The use of Hospital Episode Statistics data for the purpose evaluations of care delivered by the NHS was approved by the Confidentiality Advisory Group of the NHS Health Research Authority (15/CAG/0148). The data are anonymised and therefore their use does not require ethical approval and individual-level patient consent.

For peer review only

Funding

This study was supported by a grant from the National Institute for Health Research (NIHR) Health Services and Delivery Research (HS&DR) Programme (14/70/162). Independent scientific review of the proposal has been undertaken by NIHR. However, the authors are solely responsible for any errors or omissions as well as the opinions expressed.

For peer review only

REFERENCES

1. Hunskaar S, Lose G, Sykes D, Voss S. The prevalence of urinary incontinence in women in four European countries. BJU Int. 2004;93(3):324-30.
2. Coyne KS, Kvasz M, Ireland AM, Milsom I, Kopp ZS, Chapple CR. Urinary incontinence and its relationship to mental health and health-related quality of life in men and women in Sweden, the United Kingdom, and the United States. Eur Urol. 2012;61(1):88-95. doi: 10.1016/j.eururo.2011.07.049. Epub Jul 26.
3. Papanicolaou S, Hunskaar S, Lose G, Sykes D. Assessment of bothersomeness and impact on quality of life of urinary incontinence in women in France, Germany, Spain and the UK. BJU Int. 2005;96(6):831-8.
4. NICE. NICE clinical guideline 171: The management of urinary incontinence in women. 2013.
5. Gornall J. How mesh became a four letter word. BMJ. 2018;363:k4137.
6. Withington J, Hirji S, Sahai A. The Changing Face of Urinary Continence Surgery in England: A Perspective from the Hospital Episode Statistics Database. BJU Int. 2014.
7. NHS Digital. Retrospective Review of Surgery for Urogynaecological Prolapse and Stress Urinary Incontinence using Tape or Mesh 2018. Available from: https://digital.nhs.uk/data-and-information/publications/statistical/mesh/apr08-mar17/retrospective-review-of-surgery-for-vaginal-prolapse-and-stress-urinary-incontinence-using-tape-or-mesh-copy.
8. NHS Digital. Hospital Episode Statistics. Patient admitted care, England 2018. Available from: https://digital.nhs.uk/data-and-information/publications/statistical/hospital-admitted-patient-care-activity
9. U.S. Food & Drug Administration. FDA public health notification: serious complications associated with transvaginal placement of surgical mesh in repair of pelvic organ prolapse and stress urinary incontinence 2008.
10. NHS England. Mesh Oversight Group Report. 2017.
11. Scottish Government. Scottish Independent Review of the use, safety and efficacy of transvaginal mesh implants in the treatment of stress urinary incontinence and pelvic organ prolapse in women Edinburgh2017 [16/09/2017]. Available from: http://www.gov.scot/Resource/0051/00515856.pdf.
12. NHS England. Mesh complications. 2017.
13. Leone Roberti Maggiore U, Finazzi Agro E, Soligo M, Li Marzi V, Digesu A, Serati M. Long-term outcomes of TOT and TVT procedures for the treatment of female stress urinary incontinence: a systematic review and meta-analysis. Int Urogynecology Journal. 2017;28(8):1119-30.
14. Department of Health and Social Care. Government announces strict rules for the use of vaginal mesh 2018. Available from: https://www.gov.uk/government/news/government-announces-strict-rules-for-the-use-of-vaginal-mesh.
15. Department of Health and Social Care. Review launched to respond to patient concerns about NHS treatments 2018. Available from: https://www.gov.uk/government/news/review-launched-to-respond-to-patient-concerns-about-nhs-treatments.
16. Gibson W, Wagg A. New horizons: urinary incontinence in older people. Age Ageing. 2014;43(2):157-63.
17. Wagg A, Duckett J, McClurg D, Harari D, Lowe D. To what extent are national guidelines for the management of urinary incontinence in women adhered? Data from a national audit. BJOG: An International Journal of Obstetrics & Gynaecology. 2011;118(13):1592-600.
18. Siddiqui NY, Levin PJ, Phadtare A, Pietrobon R, Ammarell N. Perceptions about female urinary incontinence: a systematic review. Int Urogynecol J. 2013.
19. NHS Digital. OPCS-4 2018 [01/12/18]. Available from: https://hscic.kahootz.com/connect.ti/t_c_home/view?objectId=297875.
20. World Health Organization. International Statistical Classification of Diseases and Related Health Problems 10th Revision 2018 [01/12/2018]. Available from: https://icd.who.int/browse10/2016/en.

For peer review only - http://bmjopen.bmj.com/site/about/guidelines.xhtml

21. NHS England. Sustainability and Transformation Plans (STPs): Local plans to improve health and care [cited 2017 27 April]. Available from: https://www.england.nhs.uk/stps/.
22. ONS. Table 1 2011 Census: Usual resident population by five-year age group and sex, United Kingdom and constituent countries 2011.
23. Department for Communities and Local Government. The English indices of deprivation 2015. Available from: https://www.gov.uk/government/collections/english-indices-of-deprivation.
24. Greenland S. Principles of multilevel modelling. Int J Epidemiol. 2000;29(1):158-67.
25. Cromwell DA, Mahmood TA, Templeton A, van der Meulen JH. Surgery for menorrhagia within English regions: variation in rates of endometrial ablation and hysterectomy. Bjog. 2009;116(10):1373-9.
26. Bottle A, Aylin P. Variations in vaginal and abdominal hysterectomy by region and trust in England. BJOG. 2005;112(3):326-8.
27. Strickland R. Reasons for not seeking care for urinary incontinence in older community-dwelling women: a contemporary review. Urol Nurs. 2014;34(2):63-8, 94.
28. Shaw C, Tansey R, Jackson C, Hyde C, Allan R. Barriers to help seeking in people with urinary symptoms. Fam Pract. 2001;18(1):48-52.
29. Horrocks S, Somerset M, Stoddart H, Peters TJ. What prevents older people from seeking treatment for urinary incontinence? A qualitative exploration of barriers to the use of community continence services. Fam Pract. 2004;21(6):689-96.
30. Wells M, Wagg A. Integrated continence services and the female Bangladeshi population. Br J Nurs. 2007;16(9):516-9.
31. Doshani A, Pitchforth E, Mayne CJ, Tincello DG. Culturally sensitive continence care: a qualitative study among South Asian Indian women in Leicester. Fam Pract. 2007;24(6):585-93.
32. Teunissen D, van Weel C, Lagro-Janssen T. Urinary incontinence in older people living in the community: examining help-seeking behaviour. Br J Gen Pract. 2005;55(519):776-82.
33. Andersson G, Johansson JE, Nilsson K, Sahlberg-Blom E. Perceptions of urinary incontinence among Syrian Christian women living in Sweden. J Transcult Nurs. 2009;20(3):296-303.
34. Welch LC, Botelho EM, Tennstedt SL. Race and ethnic differences in health beliefs about lower urinary tract symptoms. Nurs Res. 2011;60(3):165-72.
35. Wagg A, Potter J, Peel P, Irwin P, Lowe D, Pearson M. National audit of continence care for older people: management of urinary incontinence. Age Ageing. 2008;37(1):39-44.
36. Franzen K, Andersson G, Odeberg J, Midlov P, Samuelsson E, Stenzelius K, et al. Surgery for urinary incontinence in women 65 years and older: a systematic review. Int Urogynecol J. 2015;26(8):1095-102.
37. Wennberg JE. Time to tackle unwarranted variations in practice. BMJ. 2011;342:d1513.
38. Fusco F, Abdel-Fattah M, Chapple CR, Creta M, La Falce S, Waltregny D, et al. Updated Systematic Review and Meta-analysis of the Comparative Data on Colposuspensions, Pubovaginal Slings, and Midurethral Tapes in the Surgical Treatment of Female Stress Urinary Incontinence. Eur Urol. 2017;72(4):567-91.
39. NICE. Guideline: Urinary incontinence and pelvic organ prolapse in women: managment. Draft for consultation. 2018.
40. Keltie K, Elneil S, Monga A, Patrick H, Powell J, Campbell B, et al. Complications following vaginal mesh procedures for stress urinary incontinence: an 8 year study of 92,246 women. Scientific Reports. 2017;7(1):12015.
41. Morling J, McAllister D, Agur W, Fischbacher C, Glazener C, Guerrero K, et al. Adverse events after first, single, mesh and non-mesh surgical procedures for stress urinary incontinence and pelvic organ prolapse in Scotland, 1997–2016: a population-based cohort study. The Lancet. 2017;389(10069):629-40.
42. King's Fund. The UK private health market. 2014.

For peer review only

For peer review only - http://bmjopen.bmj.com/site/about/guidelines.xhtml

List of Figures & Legends

Figure 1: Study cohort selection process
Figure Legend: Figure 1 is a flow diagram of the inclusion process for women who received surgical treatment for SUI in England. Data extracted from Hospital Episode Statistics 2013 – 2016. Abbreviations: SUI (stress urinary incontinence); ICD-10 (International Classification of Diseases, version 10) Office for Population Censuses and Surveys Classification; N39.3 (Stress urinary incontinence ICD-10 code).

Figure 2: CCG-level rates of stress urinary incontinence procedures between 2013 and 2016
Figure Legend: Figure 2 shows the empirical Bayes (EB) estimated procedure rates for stress urinary incontinence. The vertical axes in (a) and (b) are EB rates. Rates in (b) are risk-adjusted for age, socio economic status, ethnicity and long-term illness. The numbers on the horizontal axis represent the assigned position of the CCG ranked according to rates. Geographical mapping in (c) highlight the locations of CCGs with the lowest to highest range of procedure rates in England as well as an expanded section of the London Commissioning Region (CR). CCGs were not labelled because of space constraints.

Figure 3: STP-level rates of stress urinary incontinence procedures between 2013 and 2016
Figure Legend: Figure 3 shows the empirical Bayes (EB) estimated procedure rates for stress urinary incontinence. The vertical axes in (a) and (b) are EB rates. Rates in (b) are risk-adjusted for age, socio economic status, ethnicity and long-term illness. The numbers on the horizontal axis represent the assigned position of the STP footprint ranked according to rates. Geographical mapping in (c) highlight the locations of STP footprints with the lowest to highest range of procedure rates.

For peer review only

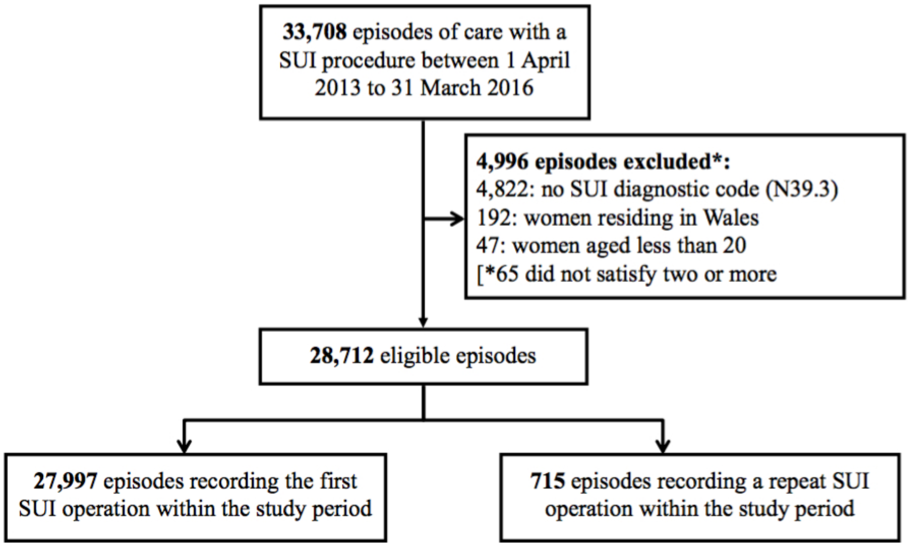

Study cohort selection process

Figure 1 is a flow diagram of the inclusion process for women who received surgical treatment for SUI in England. Data extracted from Hospital Episode Statistics 2013 – 2016. Abbreviations: SUI (stress urinary incontinence); ICD-10 (International Classification of Diseases, version 10) Office for Population Censuses and Surveys Classification; N39.3 (Stress urinary incontinence ICD-10 code).

346x209mm (72 x 72 DPI)

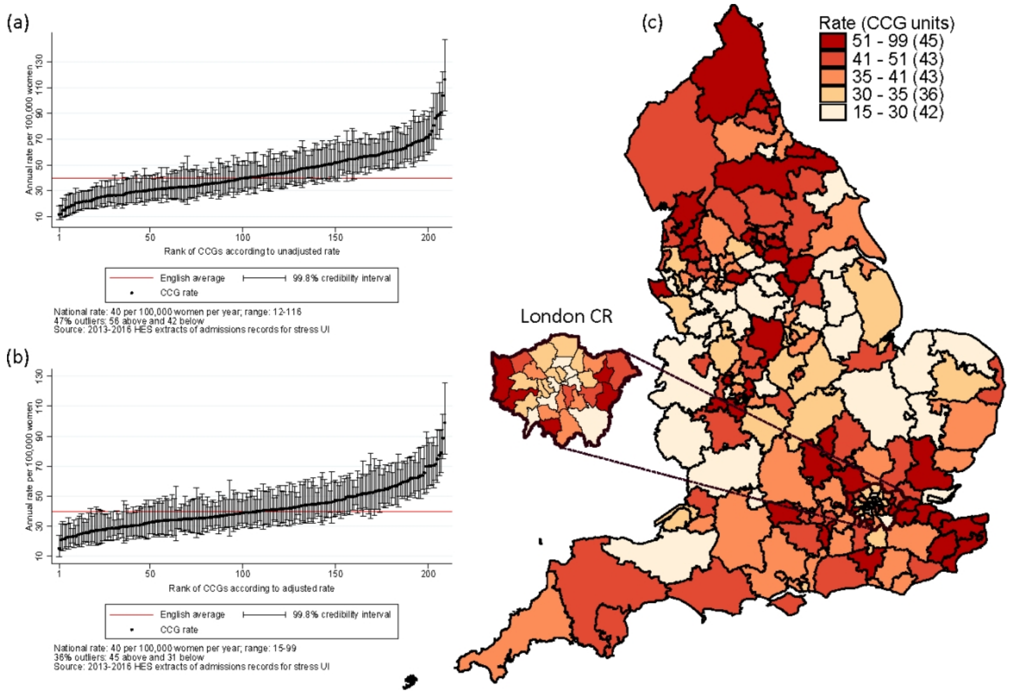

CCG-level rates of stress urinary incontinence procedures between 2013 and 2016
Figure 2 shows the empirical Bayes (EB) estimated procedure rates for stress urinary incontinence. The vertical axes in (a) and (b) are EB rates. Rates in (b) are risk-adjusted for age, socio economic status, ethnicity and long-term illness. The numbers on the horizontal axis represent the assigned position of the CCG ranked according to rates. Geographical mapping in (c) highlight the locations of CCGs with the lowest to highest range of procedure rates in England as well as an expanded section of the London Commissioning Region (CR). CCGs were not labelled because of space constraints.

165x113mm (200 x 200 DPI)

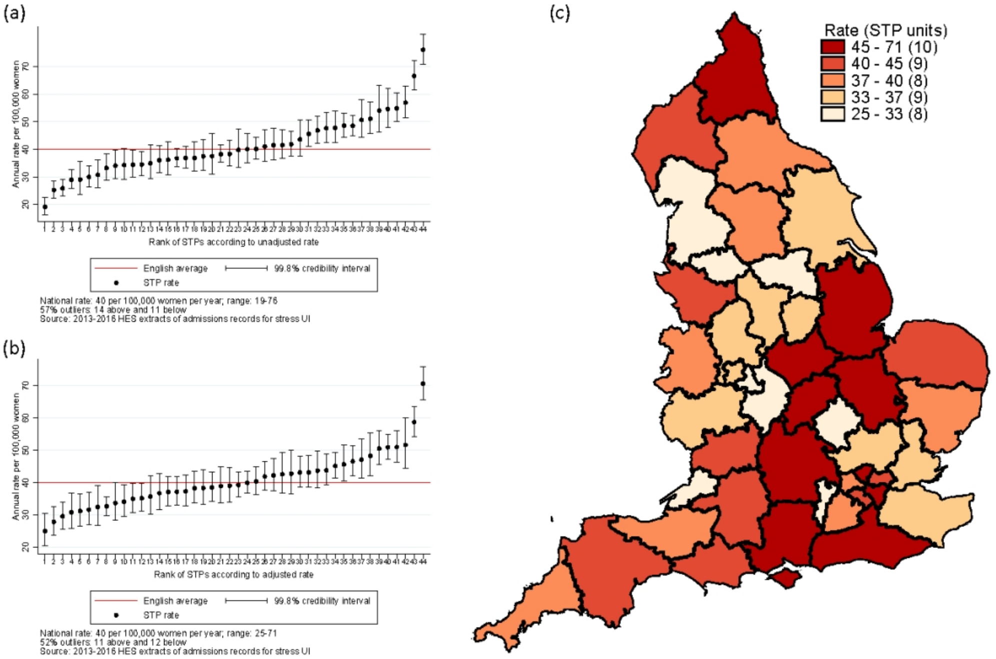

STP-level rates of stress urinary incontinence procedures between 2013 and 2016
Figure 3 shows the empirical Bayes (EB) estimated procedure rates for stress urinary incontinence. The vertical axes in (a) and (b) are EB rates. Rates in (b) are risk-adjusted for age, socio economic status, ethnicity and long-term illness. The numbers on the horizontal axis represent the assigned position of the STP footprint ranked according to rates. Geographical mapping in (c) highlight the locations of STP footprints with the lowest to highest range of procedure rates.

169x114mm (200 x 200 DPI)

STROBE Statement—Checklist of items that should be included in reports of ***cohort studies***

	Item No	Recommendation	Page Number
**Title and abstract**	1	(*a*) Indicate the study's design with a commonly used term in the title or the abstract	1
		(*b*) Provide in the abstract an informative and balanced summary of what was done and what was found	2
**Introduction**			
Background/rationale	2	Explain the scientific background and rationale for the investigation being reported	4
Objectives	3	State specific objectives, including any prespecified hypotheses	4
**Methods**			
Study design	4	Present key elements of study design early in the paper	5
Setting	5	Describe the setting, locations, and relevant dates, including periods of recruitment, exposure, follow-up, and data collection	5
Participants	6	(*a*) Give the eligibility criteria, and the sources and methods of selection of participants. Describe methods of follow-up	5
		(*b*) For matched studies, give matching criteria and number of exposed and unexposed	5
Variables	7	Clearly define all outcomes, exposures, predictors, potential confounders, and effect modifiers. Give diagnostic criteria, if applicable	5
Data sources/ measurement	8*	For each variable of interest, give sources of data and details of methods of assessment (measurement). Describe comparability of assessment methods if there is more than one group	5
Bias	9	Describe any efforts to address potential sources of bias	
Study size	10	Explain how the study size was arrived at	5
Quantitative variables	11	Explain how quantitative variables were handled in the analyses. If applicable, describe which groupings were chosen and why	5-6
Statistical methods	12	(*a*) Describe all statistical methods, including those used to control for confounding	6
		(*b*) Describe any methods used to examine subgroups and interactions	6
		(*c*) Explain how missing data were addressed	6
		(*d*) If applicable, explain how loss to follow-up was addressed	
		(*e*) Describe any sensitivity analyses	
**Results**			
Participants	13*	(a) Report numbers of individuals at each stage of study—eg numbers potentially eligible, examined for eligibility, confirmed eligible, included in the study, completing follow-up, and analysed	7 and Figure 1
		(b) Give reasons for non-participation at each stage	Figure 1
		(c) Consider use of a flow diagram	Figure 1
Descriptive data	14*	(a) Give characteristics of study participants (eg demographic, clinical, social) and information on exposures and potential confounders	Table 2
		(b) Indicate number of participants with missing data for each variable of interest	Figure 1
		(c) Summarise follow-up time (eg, average and total amount)	

For peer review only - http://bmjopen.bmj.com/site/about/guidelines.xhtml

Outcome data	15*	Report numbers of outcome events or summary measures over time	7 and Table 2
Main results	16	(*a*) Give unadjusted estimates and, if applicable, confounder-adjusted estimates and their precision (eg, 95% confidence interval). Make clear which confounders were adjusted for and why they were included	Table 2
		(*b*) Report category boundaries when continuous variables were categorized	
		(*c*) If relevant, consider translating estimates of relative risk into absolute risk for a meaningful time period	
Other analyses	17	Report other analyses done—eg analyses of subgroups and interactions, and sensitivity analyses	
**Discussion**			
Key results	18	Summarise key results with reference to study objectives	12
Limitations	19	Discuss limitations of the study, taking into account sources of potential bias or imprecision. Discuss both direction and magnitude of any potential bias	14-15
Interpretation	20	Give a cautious overall interpretation of results considering objectives, limitations, multiplicity of analyses, results from similar studies, and other relevant evidence	12-13
Generalisability	21	Discuss the generalisability (external validity) of the study results	12
**Other information**			
Funding	22	Give the source of funding and the role of the funders for the present study and, if applicable, for the original study on which the present article is based	17

*Give information separately for exposed and unexposed groups.

Note: An Explanation and Elaboration article discusses each checklist item and gives methodological background and published examples of transparent reporting. The STROBE checklist is best used in conjunction with this article (freely available on the Web sites of PLoS Medicine at http://www.plosmedicine.org/, Annals of Internal Medicine at http://www.annals.org/, and Epidemiology at http://www.epidem.com/). Information on the STROBE Initiative is available at http://www.strobe-statement.org.

For peer review only

For peer review only - http://bmjopen.bmj.com/site/about/guidelines.xhtml

BMJ Open

GEOGRAPHICAL VARIATION IN RATES OF SURGICAL TREATMENT FOR FEMALE STRESS URINARY INCONTINENCE IN ENGLAND: A NATIONAL COHORT STUDY

Journal:	*BMJ Open*
Manuscript ID	bmjopen-2019-029878.R1
Article Type:	Research
Date Submitted by the Author:	01-Jul-2019
Complete List of Authors:	Mamza, Jil; London School of Hygiene and Tropical Medicine Faculty of Public Health and Policy, Department of Health Services Research and Policy<br>Geary, Rebecca; London School of Hygiene and Tropical Medicine Faculty of Public Health and Policy, Department of Health Services Research and Policy; University College London, Department of Infection and Population Health<br>El-Hamamsy, Dina; University Hospitals of Leicester NHS Trust, Obstetrics and Gynaecology Department<br>Cromwell, David; London School of Hygiene and Tropical Medicine<br>Duckett, Jonathan; Medway NHS Foundation Trust<br>Monga, Ash ; University of Southampton<br>Toozs-Hobson, Philip; Birmingham Women's and Children's NHS Foundation Trust<br>Mahmood, Tahir; Forth Park Hospital, Obstetrics and Gynaecology<br>Wilson, Andrew; University of Leicester, Health Sciences<br>Tincello , Doug ; University of Leicester<br>van der Meulen, Jan; London School of Hygiene and Tropical Medicine<br>Gurol Urganci, Ipek; London School of Hygiene & Tropical Medicine, Health Services Research & Policy
**Primary Subject Heading**:	Health services research
Secondary Subject Heading:	Obstetrics and gynaecology
Keywords:	Urogynaecology < GYNAECOLOGY, Geographical mapping < TROPICAL MEDICINE, Minimally invasive surgery < GYNAECOLOGY

SCHOLARONE™
Manuscripts

GEOGRAPHICAL VARIATION IN RATES OF SURGICAL TREATMENT FOR FEMALE STRESS URINARY INCONTINENCE IN ENGLAND: A NATIONAL COHORT STUDY

Jil B Mamza[1*], Rebecca S Geary[1*], Dina El-Hamamsy[2], David Cromwell[1], Jonathan Duckett[3], Ash Monga[4], Philip Toozs-Hobson[5], Tahir Mahmood[6], Andrew Wilson[7], Douglas G Tincello[7], Jan van der Meulen[1#], Ipek Gurol-Urganci[1#]

[1] Department of Health Services Research and Policy, London School of Hygiene & Tropical Medicine

[2] Obstetrics and Gynaecology Department, University Hospitals of Leicester

[3] Medway NHS Foundation Trust

[4] University Hospital Southampton NHS Foundation Trust

[5] Birmingham Women's NHS Foundation Trust

[6] NHS Fife, Victoria Hospital

[7] Department of Health Sciences, University of Leicester

[*] Joint first authors have made an equal contribution to this study and manuscript

[#]Joint senior authors have made an equal contribution to this study and manuscript

Corresponding Author

Ipek Gurol-Urganci

Department of Health Services Research and Policy
London School of Hygiene & Tropical Medicine
15-17 Tavistock Place, London WC1H 9SH, United Kingdom
Telephone: +44 (0)20 7927 2266

Email: ipek.gurol@lshtm.ac.uk

Word count: 2257 words

Running title: Variation in surgery for female stress urinary incontinence

For peer review only

ABSTRACT

Objective To examine geographic variation in use of surgery for female stress urinary incontinence (SUI), mainly mid-urethral mesh tape insertions, in the English National Health Service (NHS).

Design National cohort study

Setting NHS hospitals

Participants 27,997 women aged 20 years or older who had a first stress urinary incontinence surgery in an English NHS Hospital between April 2013 and March 2016 and a diagnosis of stress urinary incontinence at the same time as the procedure

Methods Multilevel Poisson regression was used to adjust for geographic differences in age, ethnicity, prevalence of long-term illness and socio-economic deprivation.

Primary outcome measure Rate of surgery for stress urinary incontinence per 100,000 women/year at two geographic levels: Clinical Commissioning Group (CCG, n=209) and Sustainability and Transformation Partnership (STP, n=44).

Results The rate of surgery for stress urinary incontinence was 40 procedures per 100,000 women/year. Risk-adjusted rates ranged from 20 to 106 procedures per 100,000 women/year across CCGs and 24 to 69 procedures per 100,000 women/year across the STP areas. These regional differences were only partially explained by demographic characteristics as adjustment reduced variance of surgery rates by 16% among the CCGs and 35% among the STPs.

Conclusions Substantial geographic variation exists in use of surgery for female stress urinary incontinence in the English NHS, suggesting that women in some areas are more likely to be treated compared to women with the same condition in other areas. The variation reflects differences in how national guidelines are being interpreted, in the context of the ongoing debate about the safety of SUI surgery.

Funding National Institute for Health Research (NIHR) Health Services and Delivery Research (HS&DR) Programme (14/70/162)

Keywords: Stress urinary incontinence, mid-urethral mesh tape, geographical variation.

For peer review only

Article Summary

Strengths and limitations of this study

- The data used for the study includes all surgical procedures performed within English NHS Hospitals, reducing the risk of selection bias
- Statistical modelling, utilising multi-level empirical Bayes methods was used to minimise potential estimation error problems when identifying potential outlier areas
- Unmeasured confounding factors and differences in coding practices may have contributed to variation in surgery rates
- This study did not account for surgical procedures performed in private hospitals. However, it is likely that at least 90% of all continence procedures in England are provided by the NHS, as the total annual spend on private health care in England is approximately 5% of the total annual spend on the NHS.

For peer review only

For peer review only - http://bmjopen.bmj.com/site/about/guidelines.xhtml

INTRODUCTION

Urinary incontinence is estimated to affect 30 to 40% of women in the UK. (1, 2) The condition has a significant impact on quality of life (3), affecting physical and social activities, confidence and self-perception (4). Stress urinary incontinence (SUI), the involuntary loss of urine with increases in abdominal pressure such as when exercising or coughing, is the most commonly diagnosed type of incontinence in women, accounting for approximately 50% of all UI diagnoses (5). Urgency urinary incontinence (UUI) is characterised by a sudden and compelling desire to pass urine that is difficult to defer. Many women experience coexisting stress and urgency UI symptoms, a sub-type often called mixed urinary incontinence. UI is managed at the primary care level initially(6). Lifestyle changes may be recommended in primary care where women with UI also smoking cigarettes, report excessive fluid or caffeine consumption or are overweight or obese(7). Surgical treatments are recommended when conservative treatments are ineffective or not tolerated(8).

Mid-urethral mesh tapes were introduced in 1998 as a novel surgical treatment for female SUI(9). A sharp rise in the use of mesh tapes to treat SUI followed, due in part to the minimally invasive nature of the procedure, with a maximum of 11,365 procedures conducted in 2009. Over the same period, the previous standard treatment for female SUI, colposuspension (a major abdominal surgery) declined from more than 3,500 procedures per year to just 200(10). However, after the peak of more than 11,000 procedures in 2008-09, the number of mesh procedures for SUI has almost halved, falling to just 6227 by 2016-17(11, 12). The decline in the use of mesh tapes for SUI has most likely been in response to concerns about the safety of mesh(13-15) with some women experiencing pain, dyspareunia, persistent urinary incontinence, and exposure or erosion(16, 17). In 2018, the use of mesh tapes to treat SUI was suspended in the NHS in England, following an interim recommendation of the Independent Medicines and Medical Devices Safety Review(18, 19).

Previous studies highlighted that not all women with SUI have equitable access to appropriate incontinence care; access to continence surgery varies by age(20, 21) and ethnic and socio-economic backgrounds;(22) with evidence of variations in care for other

For peer review only - http://bmjopen.bmj.com/site/about/guidelines.xhtml

vulnerable populations. In light of the current suspension of the use of mid-urethral mesh tapes, the most commonly used procedures to treat female SUI, evidence is needed regarding the utilisation of mesh, and non-mesh, surgical continence procedures before the suspension was in place. A better understanding of geographical differences in access to surgical treatment for SUI in the English National Health Service (NHS) between 2013 and 2016 and of the factors contributing to this variation will be informative for future policy decisions about the appropriateness of surgical treatment of female SUI.

METHODS

Study design, setting and definitions

This study used data from Hospital Episode Statistics (HES), a routinely collected, administrative dataset which contains records of all NHS hospital admissions in England. The cohort comprised women aged 20 years and older who had received surgical treatment for SUI between 1 April 2013 and 31 March 2016 and had an SUI diagnosis recorded at the time of the procedure. SUI surgery was defined using UK Office for Population Censuses and Surveys Classification (OPCS-4) codes (Table 1)(23). SUI diagnosis was defined using the International Classification of Diseases, ICD-10 code: N39.3 Stress urinary incontinence(24). Women may have had repeat procedures in the study period, however, only the first operation was counted in calculating the rate of surgery.

Measures

The outcome measure was rate of surgery for SUI per 100,000 women/year at two geographic levels: 209 Clinical Commissioning Group (CCG) and 44 Sustainability and Transformation Partnership (STP) areas. CCGs are statutory NHS bodies responsible for the planning and commissioning of health care services in a local area (average population size of about 104,000 adult females). CCG areas are grouped into 44 STP areas (average population size of about 493,000 adult females), which were set up to coordinate improvements in the delivery of NHS services(25). Reference denominator populations were derived by aggregating the 2011 Census population counts for women aged 20 and older in lower super output areas (LSOA) that are within the respective boundaries of the CCG and

For peer review only

STP areas. LSOAs are postcode-based hierarchical geographic units designed to improve the reporting of small area statistics in England and Wales. There are 32,844 LSOAs in England with an average population approximately 1,700 people (26).

Sociodemographic factors may explain variations in rates of surgery for SUI. We handled age as a patient-level characteristic grouped into five categories (20-39, 40-49, 50-59, 60-69 and 70+ years). Reference group was chosen as 40-49 years of age as surgery for SUI is most prevalent for this age group. Socio-economic status, ethnicity and limiting long-term illness were CCG-level characteristics derived from 2011 Census data. For socio-economic status, we used the averages of the national ranking of the Index of Multiple Deprivation (IMD) (27) of LSOAs within each CCG, and grouped the CCG averages into national quintiles ranging from 1 (most deprived CCGs) to 5 (least deprived CCGs). For ethnicity, we used the percentage of the population reporting black or ethnic minority (BME) background, and for long-term illness the percentage who reported that their day-to-day activities were limited because of a health problem or disability which has lasted, or is expected to last, at least 12 months. For each CCG, we took the averages of these percentages for LSOAs and grouped these CCG averages into national quintiles ranging from 1 (CCGs with average percentages in the lowest quintile) to 5 (highest quintile).

Statistical analyses

We calculated the number and the unadjusted and adjusted rates per 100,000 women/year of SUI procedures overall, and according to patient and regional characteristics. Incidence rate ratios were used to represent associations between the procedure rate and regional characteristics. Multilevel Poisson regression models were used to produce empirical Bayes estimates of the unadjusted and adjusted incidence rates for each CCG and STP area. In addition, risk adjusted regression models were used to assess geographic variation in rates of surgery by year. The empirical Bayes estimator produces more precise results by “pulling” the estimates for small outlier regions towards the overall mean(28). For each area level (CCG/STP), we illustrated the amount of variation in adjusted surgery rates using maps and range plots with 99.8% credibility intervals. CCGs and STPs were marked as “outliers” where

For peer review only

the national average rate of surgery was not within the 99.8% credibility interval of their rates. All statistical calculations were performed using Stata 14.

Patient involvement

This study was supported by a steering group which included lay members and patient representatives who provided input to the design of the study and interpretation of the results and contributed to the dissemination plan. The steering group met on a regular basis for the duration of the study.

RESULTS

Description of the cohort

Between April 2013 and March 2016, there were 33,708 inpatient episodes with a surgical procedure for SUI. 4,996 of these episodes did not satisfy the inclusion criteria, for example, because they did not have an SUI diagnosis recorded at the time of the procedure, outlined in Figure 1, and 715 recorded a subsequent operation in the study period (Figure 1). 27,997 procedures were included in the analyses, 90% of which were mid-urethral mesh tape insertions (Table 1). Restricting our analyses to these 27,997 first SUI procedures captured >97% of all 28,789 SUI procedures in the study period, and the distribution of procedure types did not vary between all and first procedures (Table 1). The national annual rate of surgery was 40 procedures per 100,000 women.

For peer review only

Table 1: OPCS-4 codes and counts of SUI procedures with relevant diagnosis (ICD-10) code N39.3

OPSC-4	Description	All operations* N (%)	First operations** N (%)
	**Mid-urethral tape insertions**		
M53.3	Introduction of tension-free vaginal tape	16665 (57.9)	16415 (58.6)
M53.6	Introduction of transobturator tape	8866 (30.8)	8722 (31.2)
	**Injection of urethral bulking agents**		
M56.3	Endoscopic injection of inert substance into outlet of female bladder	1628 (5.7)	1435 (5.1)
	**Other abdominal / vaginal operations**		
M51.1	Abdominoperineal suspension of urethra	32 (0.1)	29 (0.1)
M51.2	Endoscopic suspension of neck of bladder	6 (<0.1)	6 (<0.1)
M51.8	Other specified combined abdominal and vaginal operations to support outlet of female bladder	15 (0.1)	13 (<0.1)
M51.9	Unspecified combined abdominal and vaginal operations to support outlet of female bladder	2 (<0.1)	2 (<0.1)
M52.1	Suprapubic sling operation	355 (1.2)	328 (1.2)
M52.2	Retropubic suspension of neck of bladder	78 (0.3)	76 (0.3)
M52.3	Colposuspension of neck of bladder	587 (2.0)	533 (1.9)
M52.8	Other specified abdominal operations to support outlet of female bladder	20 (0.1)	15 (0.1)
M52.9	Unspecified abdominal operations to support outlet of female bladder	3 (<0.1)	2 (<0.1)
M53.1	Vaginal buttressing of urethra	130 (0.5)	126 (0.5)
M53.8	Other specified vaginal operations to support outlet of female bladder	302 (1.0)	216 (0.8)
M53.9	Unspecified vaginal operations to support outlet of female bladder	5 (<0.1)	4 (<0.1)
M55.2	Implantation of artificial urinary sphincter into outlet of female bladder	18 (0.1)	11 (<0.1)
M55.6	Insertion of retropubic device for female stress urinary incontinence NEC	56 (0.2)	52 (0.2)
M55.8	Other specified other open operations on outlet of female bladder	14 (<0.1)	8 (<0.1)
M55.9	Unspecified other open operations on outlet of female bladder	0 (0)	0 (0)
M58.8	Other specified other operations on outlet of female bladder	7 (<0.1)	4 (<0.1)
M58.9	Unspecified other operations on outlet of female bladder	0 (0)	0 (0)
	**Total**	28789	27997

Abbreviations: SUI (stress urinary incontinence); ICD-10 (International Classification of Diseases, version 10); OPCS (Office for Population Censuses and Surveys Classification); N39.3 (Stress urinary incontinence ICD-10 code). *For 77 episodes of care out of 28712 eligible episodes, two procedures were recorded and both are included in the overall count. ** For episodes of care where two procedures were recorded, only the more invasive or specified procedure is counted as first operation.

For peer review only

For peer review only - http://bmjopen.bmj.com/site/about/guidelines.xhtml

Geographic variations in surgery

Figures 2a and 3a show the variation in the unadjusted empirical Bayes estimates of the procedure rates for SUI across the CCGs and STPs, with figures 2b and 3b illustrating the rates adjusted for patients' age and the CCG-level characteristics: socio-economic status, percentage of the population reporting BME background, and percentage with a long-term illness. Figures 2c and 3c highlight the locations of CCGs/STPs with the lowest to highest range of procedure rates in England.

The adjusted SUI procedure rates for CCGs ranged from 20 to 106 procedures compared with unadjusted rates of 11 to 120 procedures per 100,000 women/year (Figure 2). Ninety-nine CCGs (47%) were marked as “outliers” (where the national average was not within the 99.8% credibility interval of their rate). These comprised 43 CCGs (20.6%) with rates below the national average and 56 CCGs (26.8%) with rates above the national average. Risk adjustment reduced the number of CCGs marked as “outliers” from 99 (47.4%) to 75 (36%). The standard deviation (SD) of the CCG-level variation in adjusted rates (SD 0.27, 95%CI: 0.24-0.30) was 16% lower than the SD of the unadjusted rates (SD 0.32, 95%CI: 0.29-0.36).

The adjusted SUI procedure rates for STPs ranged from 24 to 69 procedures compared with unadjusted rates of 20 to 77 per 100 000 women/year (Figure 3). Risk adjustment reduced the number of STPs identified as outliers from 23 (52%) to 22 (50%). The amount of variation observed declined by 35% after risk adjustment: the SD of the STP-level variation for unadjusted and adjusted model were 0.23 (95%CI: 0.17-0.31) and 0.15 (95%CI: 0.11-0.22) respectively.

Annual SUI procedure rates declined over the study period from 52 per 100,000 women in 2013 to 36 per 100,000 women in 2015. However, there was no evidence that CCG- or STP-level variation changed over time. In separate (adjusted) regression models run by year, the SD of CCG-level variation was 0.26 (95%CI: 0.23-0.30) in 2013; 0.27 (95%CI: 0.23-0.31) in 2014 and 0.29 (95%CI: 0.25-0.34) in 2015. The SD of STP-level variation (adjusted model) was 0.13 (95%CI: 0.08-0.20) in 2013, 0.17 (95%CI: 0.11-0.25) in 2014 and 0.18 (95%CI: 0.12-0.26) in 2015.

For peer review only - http://bmjopen.bmj.com/site/about/guidelines.xhtml

Association of patient and regional characteristics with surgery rates

Table 2 shows the rates of surgery by regional characteristics. Rates were lowest for the 20-39 year age group (16 per 100,000 women/year), and highest for 40-49 year age group (84 per 100,000 women/year), declining with age beyond 50 years. Compared to the rate among women aged 40-49 years, the surgery rate for women aged 50-59 years was 20% lower (IRR 0.80, 95% CI 0.78-0.83), for women aged 60 to 69 years was 46% lower (IRR 0.54, 95% CI 0.52-0.56), and for women aged 70+ years was 69% lower (IRR 0.31, 95% CI 0.30-0.33).

Rates of surgery were lower for areas with higher proportions of BME populations (highest vs lowest quintile IRR 0.63, 95% CI 0.49-0.81). There were no differences in surgery rates according to the proportion of people with long-term limiting illness or socioeconomic deprivation at the CCG-level.

For peer review only

Table 2: Regional characteristics and their association with SUI procedure rates

Regional factor	Scale of factor (1 unit)	Procedures, n (%)	Crude rate per 100,000 women/year	Procedure rate ratio (95%CI)	*P* value*
**Age categories**					
20-39	Age group in years	3253 (11.6)	15.9	0.18 (0.17-0.19)	
40-49		9761 (34.9)	84.4	Reference	<0.001
50-59		7496 (26.8)	67.5	0.80 (0.78-0.83)	
60-69		4352 (15.5)	46.2	0.54 (0.52-0.56)	
≥70		3135 (11.2)	26.8	0.31 (0.30-0.33)	
**Socio-economic status**					
Most deprived	Quintile category of IMD ranking	5838 (20.9)	43.0	Reference	0.84
More deprived		6315 (22.6)	47.5	1.08 (0.93-1.25)	
Average		6371 (22.8)	47.9	1.05 (0.89-1.25)	
Less deprived		5001 (17.9)	39.9	1.02 (0.85-1.21)	
Least deprived		4472 (15.1)	36.3	1.05 (0.85-1.29)	
**Black and minority ethnic (BME) population**					
1: CCGs with lowest proportion	Ranked category of proportion of BME population	5579 (19.9)	48.8	Reference	0.001
2		6867 (24.5)	49.8	1.02 (0.89-1.17)	
3		6326 (22.6)	45.7	1.00 (0.86-1.17)	
4		5725 (20.4)	41.5	0.89 (0.75-1.06)	
5: CCGs with highest proportion		3500 (12.5)	27.2	0.63 (0.49-0.81)	
**Limiting long-term illness**					
1: CCGs with lowest proportion	Ranked category of proportion of people with limiting illness	4433 (15.8)	32.8	Reference	0.46
2		6328 (22.6)	44.4	1.16 (0.99-1.36)	
3		4882 (17.4)	43.7	1.11 (0.91-1.34)	
4		6896 (24.6)	46.1	1.12 (0.91-1.39)	
5: CCGs with highest proportion		5458 (19.5)	48.9	1.16 (0.91-1.49)	

Random effects estimates	Standard deviation† (95%CI)	Standard deviation‡ (95%CI)
STP-level variation (level 2)	0.23 (0.17-0.31)	0.15 (0.11-0.22)
CCG-level variation (level 1)	0.32 (0.29-0.36)	0.27 (0.24-0.30)

Table 2 describes the distribution of regional characteristics and the association between these factors and SUI procedure rates from the multilevel random-intercept Poisson regression model. Abbreviations: CI (Confidence interval); IMD (Index of multiple deprivation); CCG (Clinical Commissioning Group); STP (Sustainability and Transformation Plan); NHS (National Health Service); †Unadjusted estimates; ‡Adjusted for all regional factors including ethnicity. *P value obtained from likelihood ratio test.

For peer review only - http://bmjopen.bmj.com/site/about/guidelines.xhtml

DISCUSSION

Main findings

More than 30,000 women were admitted to NHS hospitals in England for an SUI-related surgical treatment between April 2013 and March 2016. The rate of surgery for SUI was 40 procedures per 100,000 women/year. Crude regional rates varied by a factor of eleven among the 209 CCGs from 11 to 120, and a factor of four among 44 STPs from 20 to 77 procedures per 100,000 women/year. These differences were only slightly reduced when the women's age and regional characteristics were taken into account. The overall rate of SUI surgery dropped by a third over the three-year study period whilst the extent of geographic variation remained stable.

Interpretation

This study, carried out in the English NHS, is the first national study to explore geographic variation in rates of surgical treatment for SUI. Evidence to date regarding geographic variation in benign gynaecological surgery across England focused primarily on surgery for menorrhagia(29, 30) suggesting substantial variation despite the existence of national guidelines.

We found that women's age and regional ethnicity distributions were associated with surgery rates. This may reflect differences in incontinence-related health beliefs, preferences and care seeking behaviour for older women(31) and women from various ethnic backgrounds(22) or inequitable use of surgical care (32) . Studies suggest that only around half of older people seek help for their incontinence symptoms, commonly due to the belief that it is a normal part of ageing.(33, 34) In England, studies concluded that help-seeking behaviour was hindered for South-Asian women as they felt embarrassed to discuss sensitive problems, particularly with a male health professional.(35, 36) Other studies in the Netherlands,(37) Sweden(38) and the USA(39) also found notable differences in preferences across women from different age groups and ethnic backgrounds.

For peer review only - http://bmjopen.bmj.com/site/about/guidelines.xhtml

We found that older women were less likely to have received surgical treatment for their SUI. This agrees with findings for other aspects of continence care. A national audit for continence care in the UK(20) found that deficiencies in the organisation of care and the management of urinary incontinence were more pronounced for older people.(40) For example, in acute and primary care settings, older people were less likely to have a continence history or focused examination done. In secondary care, whilst it has been shown that surgical treatments are safe and effective in older women,(41) these procedures were used less frequently than in younger patients(20 , 42).

In their work on clinical practice variation, Wennberg and colleagues emphasise three factors as possible sources of variation: clinical uncertainty about the appropriateness of care, regional differences in patients' preferences for particular treatments , and differences in the capacity or supply of services(43). In the context of SUI surgery, a part of the observed variation will reflect the ongoing debate and concerns about the safety of mid-urethral mesh tape procedures for women with SUI, which in 2018 led to a 'pause' in the use of mesh for the treatment of stress urinary incontinence(13-15, 18, 19). It is important to note that adjustment for factors that are likely to affect patients' preferences had little impact on the geographic variation we observed for SUI surgery. However, patients' preferences will also be strongly guided by the advice received from their clinicians. The geographic areas used in this study (CCGs and STPs) are defined by NHS bodies that commission local hospital services which suggests that differences in capacity of the local healthcare system may have contributed to the observed variation.

The "correct" rate of SUI surgery is difficult to determine, especially given the ongoing concerns about the safety of mesh tapes. However, with the observed level of geographic variation, it is likely that women in some areas were more likely to be treated compared to women with the same condition in other areas. Informed patient choice, shared decision making and improved communication of the risks and benefits of both mesh and non-mesh procedures(44) is often proposed as a possible solution(43). NICE, the organisation that develops clinical guidelines for the English NHS, recommends a multidisciplinary team review prior to offering invasive therapy for SUI symptoms(8). In light of recent reviews, and the current suspension of mesh tape

For peer review only

For peer review only - http://bmjopen.bmj.com/site/about/guidelines.xhtml

insertions, NICE's latest draft guidance (published October 2018) also states that non-surgical options for SUI must be offered before any surgical treatment(45). A better understanding of relevant, long-term clinical outcomes is also needed(46, 47). With the current level of uncertainty about the safety and outcomes of mid-urethral mesh tape insertions, it is likely that the geographic variation we observed will continue.

In England, discussions are ongoing about setting up a national prospective registry of mid-urethral mesh tape insertions to monitor reoperations and removals as outcomes. However, there is also a clear need to capture a wider range of clinical outcomes that are directly relevant to women, including recurrent or persistent urinary incontinence, pain, and sexual dysfunction. These types of outcomes can only be collected if women themselves are actively involved in the process.

Strengths and limitations

The data used comprised information on all surgical procedures performed within the English NHS, thereby reducing the risk of selection bias. Our statistical modelling, utilising multi-level empirical Bayes methods allowed for minimising potential estimation error problems and taking account of area size variations for estimation of credibility intervals(28). This approach provided a powerful and statistically robust basis for identifying potential outlier areas.

Our analyses were subject to limitations inherent to observational studies. Firstly, unmeasured confounding factors may have contributed to variation in surgery rates. We were unable to account for potential regional variation in the average severity of the SUI problems. Secondly, whilst the overall quality of clinical information in HES has been found to be sufficiently high for research and audit purposes, inaccuracies in coding practices could have introduced some variation between geographic areas. Finally, we were unable to account for procedures done in the private sector. Although precise figures are lacking, it is likely that at least 90% of all incontinence procedures carried out in England are provided by the NHS, given that the total annual spending on private health care in England is about 5% of the total annual spending on

For peer review only - http://bmjopen.bmj.com/site/about/guidelines.xhtml

For peer review only

the NHS(48). While our study findings must be interpreted with caution in light of the above limitations, these are very unlikely to explain the large regional differences observed.

CONCLUSION

We found substantial variation in the rates of surgical treatment for female SUI between geographic regions across England. Adjusting for the women's age group and regional characteristics reduced variation only slightly. It is likely that the observed variation is in part linked to the ongoing debate about the safety of mid-urethral mesh tapes leading to differences in professional opinion about the appropriateness of surgical treatment for female SUI. This can only be informed by large-scale national studies monitoring long-term outcomes relevant to patients.

For peer review only

Acknowledgements

We would like to thank Lynn Copley and Natalie Eugene at the Clinical Effectiveness Unit of the Royal College of Surgeons of England and London School of Hygiene and Tropical Medicine for extracting the required data.

Disclosure of Interests

All authors received grant funding from the National Institute for Health Research (NIHR) Health Services and Delivery Research (HS&DR) Programme (14/70/162) during the conduct of the study. AM reports personal fees from: Astellas, Pfizer, Contura and Atlantic Medical; and PTH reports personal fees from: Boston Scientifc and Ethicon, and grants and personal fees from SEP Pharma outside the submitted work.

Contribution to Authorship

The study was conceived and designed by all authors (JBM, RSG, DEH, DC, JD, AM, PTH, TM, AW, DGT, JvdM, IGU). JBM and IGU organised the datasets and performed the statistical analysis, JBM wrote the first draft of the manuscript; RSG, IGU, JBM and JvdM wrote the final manuscript, with input from DEH, DC, JD, AM, PTH, TM, AW, DGT. All authors (JBM, RSG, DEH, DC, JD, AM, PTH, TM, AW, DGT, JvdM, IGU) contributed to the interpretation of results and approved the final text. Joint senior authors (IGU and JvdM) and joint first authors (JBM and RSG) made an equal contribution to this study and manuscript.

Patient Consent and Ethics Approval

The use of Hospital Episode Statistics data for the purpose evaluations of care delivered by the NHS was approved by the Confidentiality Advisory Group of the NHS Health Research Authority (15/CAG/0148). The data are anonymised and therefore their use does not require ethical approval and individual-level patient consent.

For peer review only

Funding

This study was supported by a grant from the National Institute for Health Research (NIHR) Health Services and Delivery Research (HS&DR) Programme (14/70/162). Independent scientific review of the proposal has been undertaken by NIHR. However, the authors are solely responsible for any errors or omissions as well as the opinions expressed.

Data availability

Hospital Episode Statistics data are available on application from NHS Digital**.**

For peer review only

For peer review only - http://bmjopen.bmj.com/site/about/guidelines.xhtml

REFERENCES

1. Hunskaar S, Lose G, Sykes D, Voss S. The prevalence of urinary incontinence in women in four European countries. BJU international. 2004;93(3):324-30.
2. McGrother CW, Donaldson MM, Shaw C, Matthews RJ, Hayward TA, Dallosso HM, et al. Storage symptoms of the bladder: prevalence, incidence and need for services in the UK. BJU international. 2004;93(6):763-9.
3. Coyne KS, Kvasz M, Ireland AM, Milsom I, Kopp ZS, Chapple CR. Urinary incontinence and its relationship to mental health and health-related quality of life in men and women in Sweden, the United Kingdom, and the United States. Eur Urol. 2012;61(1):88-95. doi: 10.1016/j.eururo.2011.07.049. Epub Jul 26.
4. Papanicolaou S, Hunskaar S, Lose G, Sykes D. Assessment of bothersomeness and impact on quality of life of urinary incontinence in women in France, Germany, Spain and the UK. BJU international. 2005;96(6):831-8.
5. Minassian VA, Drutz HP, Al-Badr A. Urinary incontinence as a worldwide problem. Int J Gynaecol Obstet. 2003;82(3):327-38.
6. Monz B, Hampel C, Porkess S, Wagg A, Pons ME, Samsioe G, et al. A description of health care provision and access to treatment for women with urinary incontinence in Europe -- a five-country comparison. Maturitas. 2005;52 Suppl 2:S3-12.
7. Lukacz ES, Santiago-Lastra Y, Albo ME, Brubaker L. Urinary Incontinence in Women: A Review. JAMA : the journal of the American Medical Association. 2017;318(16):1592-604.
8. NICE. NICE clinical guideline 171: The management of urinary incontinence in women. 2013.
9. Gornall J. How mesh became a four letter word. Bmj. 2018;363:k4137.
10. Withington J, Hirji S, Sahai A. The Changing Face of Urinary Continence Surgery in England: A Perspective from the Hospital Episode Statistics Database. BJU international. 2014.
11. NHS Digital. Retrospective Review of Surgery for Urogynaecological Prolapse and Stress Urinary Incontinence using Tape or Mesh 2018 [Available from: https://digital.nhs.uk/data-and-information/publications/statistical/mesh/apr08-mar17/retrospective-review-of-surgery-for-vaginal-prolapse-and-stress-urinary-incontinence-using-tape-or-mesh-copy.
12. NHS Digital. Hospital Episode Statistics. Patient admitted care, England 2018 [Available from: https://digital.nhs.uk/data-and-information/publications/statistical/hospital-admitted-patient-care-activity
13. U.S. Food & Drug Administration. FDA public health notification: serious complications associated with transvaginal placement of surgical mesh in repair of pelvic organ prolapse and stress urinary incontinence 2008.
14. NHS England. Mesh Oversight Group Report. 2017.
15. Scottish Government. Scottish Independent Review of the use, safety and efficacy of transvaginal mesh implants in the treatment of stress urinary incontinence and pelvic organ prolapse in women Edinburgh2017 [Available from: http://www.gov.scot/Resource/0051/00515856.pdf.
16. NHS England. Mesh complications. 2017.
17. Leone Roberti Maggiore U, Finazzi Agro E, Soligo M, Li Marzi V, Digesu A, Serati M. Long-term outcomes of TOT and TVT procedures for the treatment of female stress urinary incontinence: a systematic review and meta-analysis. Int Urogynecology Journal. 2017;28(8):1119-30.
18. Department of Health and Social Care. Government announces strict rules for the use of vaginal mesh 2018 [Available from: https://www.gov.uk/government/news/government-announces-strict-rules-for-the-use-of-vaginal-mesh.
19. Department of Health and Social Care. Review launched to respond to patient concerns about NHS treatments 2018 [Available from: https://www.gov.uk/government/news/review-launched-to-respond-to-patient-concerns-about-nhs-treatments.
20. Wagg A, Duckett J, McClurg D, Harari D, Lowe D. To what extent are national guidelines for the management of urinary incontinence in women adhered? Data from a national audit. BJOG: An International Journal of Obstetrics & Gynaecology. 2011;118(13):1592-600.

For peer review only

21. Gibson W, Wagg A. Are older women more likely to receive surgical treatment for stress urinary incontinence since the introduction of the mid-urethral sling? An examination of Hospital Episode Statistics data. BJOG : an international journal of obstetrics and gynaecology. 2016;123(8):1386-92.
22. Siddiqui NY, Levin PJ, Phadtare A, Pietrobon R, Ammarell N. Perceptions about female urinary incontinence: a systematic review. International urogynecology journal. 2013.
23. NHS Digital. OPCS-4 2018 [Available from: https://hscic.kahootz.com/connect.ti/t_c_home/view?objectId=297875.
24. World Health Organization. International Statistical Classification of Diseases and Related Health Problems 10th Revision 2018 [Available from: https://icd.who.int/browse10/2016/en.
25. NHS England. Sustainability and Transformation Plans (STPs): Local plans to improve health and care [Available from: https://www.england.nhs.uk/stps/.
26. ONS. Table 1 2011 Census: Usual resident population by five-year age group and sex, United Kingdom and constituent countries 2011.
27. Department for Communities and Local Government. The English indices of deprivation 2015 [Available from: https://www.gov.uk/government/collections/english-indices-of-deprivation.
28. Greenland S. Principles of multilevel modelling. Int J Epidemiol. 2000;29(1):158-67.
29. Cromwell DA, Mahmood TA, Templeton A, van der Meulen JH. Surgery for menorrhagia within English regions: variation in rates of endometrial ablation and hysterectomy. BJOG : an international journal of obstetrics and gynaecology. 2009;116(10):1373-9.
30. Bottle A, Aylin P. Variations in vaginal and abdominal hysterectomy by region and trust in England. BJOG : an international journal of obstetrics and gynaecology. 2005;112(3):326-8.
31. Strickland R. Reasons for not seeking care for urinary incontinence in older community-dwelling women: a contemporary review. Urologic nursing. 2014;34(2):63-8, 94.
32. Geary RS, Gurol-Urganci I, Kiran A, Cromwell DA, Bansi-Matharu L, Shakespeare J, et al. Factors associated with receiving surgical treatment for menorrhagia in England and Wales: findings from a cohort study of the National Heavy Menstrual Bleeding Audit. BMJ open. 2019;9(2):e024260.
33. Shaw C, Tansey R, Jackson C, Hyde C, Allan R. Barriers to help seeking in people with urinary symptoms. Family practice. 2001;18(1):48-52.
34. Horrocks S, Somerset M, Stoddart H, Peters TJ. What prevents older people from seeking treatment for urinary incontinence? A qualitative exploration of barriers to the use of community continence services. Family practice. 2004;21(6):689-96.
35. Wells M, Wagg A. Integrated continence services and the female Bangladeshi population. British journal of nursing. 2007;16(9):516-9.
36. Doshani A, Pitchforth E, Mayne CJ, Tincello DG. Culturally sensitive continence care: a qualitative study among South Asian Indian women in Leicester. Family practice. 2007;24(6):585-93.
37. Teunissen D, van Weel C, Lagro-Janssen T. Urinary incontinence in older people living in the community: examining help-seeking behaviour. The British journal of general practice : the journal of the Royal College of General Practitioners. 2005;55(519):776-82.
38. Andersson G, Johansson JE, Nilsson K, Sahlberg-Blom E. Perceptions of urinary incontinence among Syrian Christian women living in Sweden. J Transcult Nurs. 2009;20(3):296-303.
39. Welch LC, Botelho EM, Tennstedt SL. Race and ethnic differences in health beliefs about lower urinary tract symptoms. Nurs Res. 2011;60(3):165-72.
40. Wagg A, Potter J, Peel P, Irwin P, Lowe D, Pearson M. National audit of continence care for older people: management of urinary incontinence. Age and ageing. 2008;37(1):39-44.
41. Franzen K, Andersson G, Odeberg J, Midlov P, Samuelsson E, Stenzelius K, et al. Surgery for urinary incontinence in women 65 years and older: a systematic review. International urogynecology journal. 2015;26(8):1095-102.
42. Gibson W, Wagg A. New horizons: urinary incontinence in older people. Age and ageing. 2014;43(2):157-63.
43. Wennberg JE. Time to tackle unwarranted variations in practice. Bmj. 2011;342:d1513.

For peer review only

44. Fusco F, Abdel-Fattah M, Chapple CR, Creta M, La Falce S, Waltregny D, et al. Updated Systematic Review and Meta-analysis of the Comparative Data on Colposuspensions, Pubovaginal Slings, and Midurethral Tapes in the Surgical Treatment of Female Stress Urinary Incontinence. European urology. 2017;72(4):567-91.
45. NICE. Guideline: Urinary incontinence and pelvic organ prolapse in women: managment. Draft for consultation. 2018.
46. Keltie K, Elneil S, Monga A, Patrick H, Powell J, Campbell B, et al. Complications following vaginal mesh procedures for stress urinary incontinence: an 8 year study of 92,246 women. Scientific Reports. 2017;7(1):12015.
47. Morling J, McAllister D, Agur W, Fischbacher C, Glazener C, Guerrero K, et al. Adverse events after first, single, mesh and non-mesh surgical procedures for stress urinary incontinence and pelvic organ prolapse in Scotland, 1997–2016: a population-based cohort study. The Lancet. 2017;389(10069):629-40.
48. King's Fund. The UK private health market. 2014.

For peer review only

List of Figures & Legends

Figure 1: Study cohort selection process
Figure Legend: Figure 1 is a flow diagram of the inclusion process for women who received surgical treatment for SUI in England. Data extracted from Hospital Episode Statistics 2013 – 2016. Abbreviations: SUI (stress urinary incontinence); ICD-10 (International Classification of Diseases, version 10) Office for Population Censuses and Surveys Classification; N39.3 (Stress urinary incontinence ICD-10 code).

Figure 2: CCG-level rates of stress urinary incontinence procedures between 2013 and 2016
Figure Legend: Figure 2 shows the empirical Bayes (EB) estimated procedure rates for stress urinary incontinence. The vertical axes in (a) and (b) are EB rates. Rates in (b) are risk-adjusted for age, socio economic status, ethnicity and long-term illness. The numbers on the horizontal axis represent the assigned position of the CCG ranked according to rates. Geographical mapping in (c) highlight the locations of CCGs with the lowest to highest range of procedure rates in England as well as an expanded section of the London Commissioning Region (CR). CCGs were not labelled because of space constraints.

Figure 3: STP-level rates of stress urinary incontinence procedures between 2013 and 2016
Figure Legend: Figure 3 shows the empirical Bayes (EB) estimated procedure rates for stress urinary incontinence. The vertical axes in (a) and (b) are EB rates. Rates in (b) are risk-adjusted for age, socio economic status, ethnicity and long-term illness. The numbers on the horizontal axis represent the assigned position of the STP footprint ranked according to rates. Geographical mapping in (c) highlight the locations of STP footprints with the lowest to highest range of procedure rates.

For peer review only

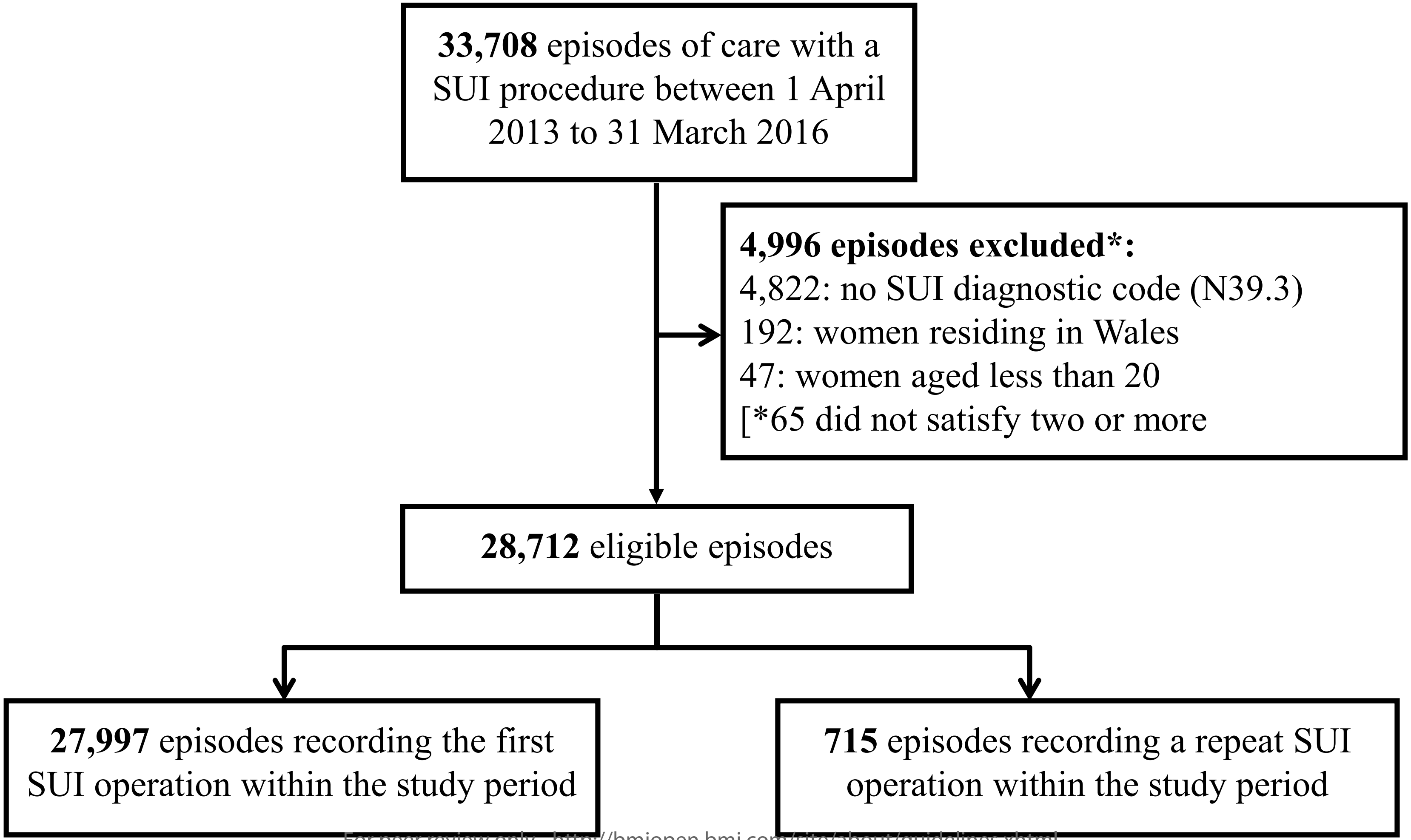

For peer review only - http://bmjopen.bmj.com/site/about/guidelines.xhtml

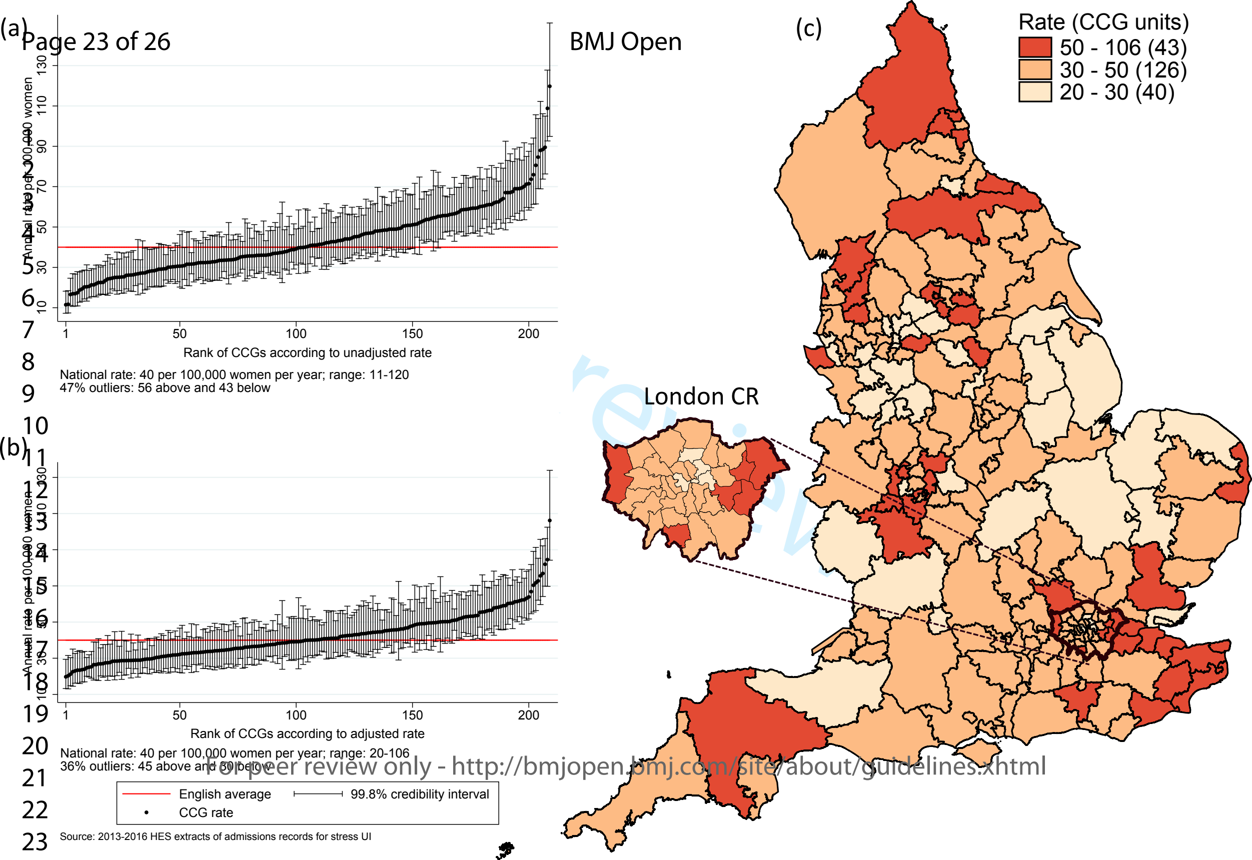

For peer review only - http://bmjopen.bmj.com/site/about/guidelines.xhtml

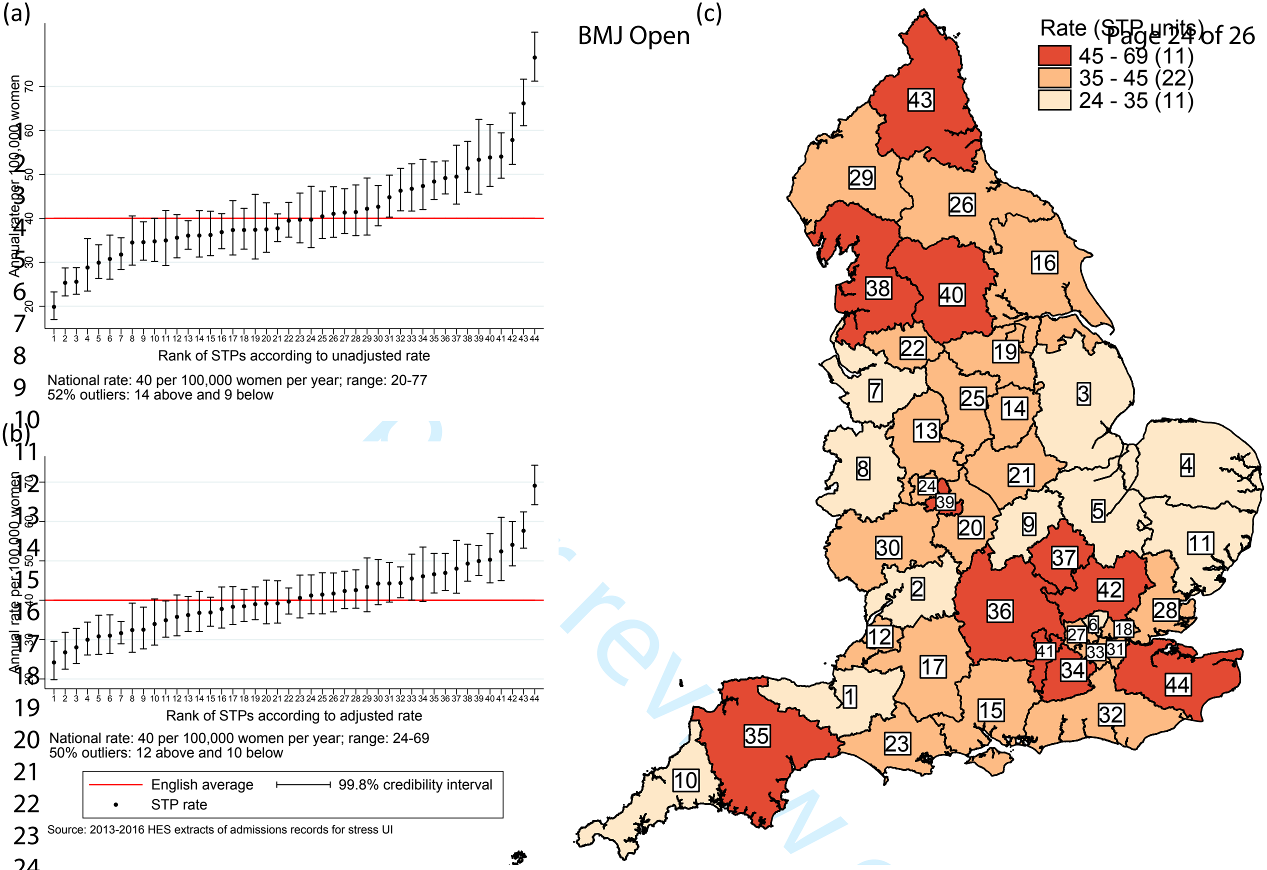

Key

1.Somerset
2.Gloucestershire
3.Lincolnshire
4.Norfolk and Waveney
5.Cambridgeshire and Peterborough
6.North Central London
7.Cheshire and Merseyside
8.Shropshire and Telford and Wrekin
9.Northamptonshire
10.Cornwall and the Isles of Scilly
11.Suffolk and North East Essex
12.Bristol, North Somerset and South Gloucestershire
13.Staffordshire
14.Nottinghamshire
15.Hampshire and the Isle of Wight
16.Coast, Humber and Vale
17.Bath, Swindon and Wiltshire
18.North East London
19.South Yorkshire and Bassetlaw
20.Coventry and Warwickshire
21.Leicester, Leicestershire and Rutland
22.Greater Manchester
23.Dorset
24.The Black Country
25.Derbyshire
26.Durham, Darlington, Teesside, Hambleton, Richmondshire and Whitby
27.North West London
28.Mid and South Essex
29.West, North and East Cumbria
30.Herefordshire and Worcestershire
31.South East London
32.Sussex and East Surrey
33.South West London
34.Surrey Heartlands
35.Devon
36.Buckinghamshire, Oxfordshire and Berkshire West
37.Milton Keynes, Bedfordshire and Luton
38.Lancashire and South Cumbria
39.Birmingham and Solihull
40.West Yorkshire
41.Frimley Health
42.Hertfordshire and West Essex
43.Northumberland, Tyne and Wear and North Durham
44.Kent and Medway

STROBE Statement—Checklist of items that should be included in reports of ***cohort studies***

	Item No	Recommendation	Page Number
**Title and abstract**	1	(*a*) Indicate the study's design with a commonly used term in the title or the abstract	1
		(*b*) Provide in the abstract an informative and balanced summary of what was done and what was found	2
**Introduction**			
Background/rationale	2	Explain the scientific background and rationale for the investigation being reported	4
Objectives	3	State specific objectives, including any prespecified hypotheses	4
**Methods**			
Study design	4	Present key elements of study design early in the paper	5
Setting	5	Describe the setting, locations, and relevant dates, including periods of recruitment, exposure, follow-up, and data collection	5
Participants	6	(*a*) Give the eligibility criteria, and the sources and methods of selection of participants. Describe methods of follow-up	5
		(*b*) For matched studies, give matching criteria and number of exposed and unexposed	5
Variables	7	Clearly define all outcomes, exposures, predictors, potential confounders, and effect modifiers. Give diagnostic criteria, if applicable	5
Data sources/ measurement	8*	For each variable of interest, give sources of data and details of methods of assessment (measurement). Describe comparability of assessment methods if there is more than one group	5
Bias	9	Describe any efforts to address potential sources of bias	
Study size	10	Explain how the study size was arrived at	5
Quantitative variables	11	Explain how quantitative variables were handled in the analyses. If applicable, describe which groupings were chosen and why	5-6
Statistical methods	12	(*a*) Describe all statistical methods, including those used to control for confounding	6
		(*b*) Describe any methods used to examine subgroups and interactions	6
		(*c*) Explain how missing data were addressed	6
		(*d*) If applicable, explain how loss to follow-up was addressed	
		(*e*) Describe any sensitivity analyses	
**Results**			
Participants	13*	(a) Report numbers of individuals at each stage of study—eg numbers potentially eligible, examined for eligibility, confirmed eligible, included in the study, completing follow-up, and analysed	7 and Figure 1
		(b) Give reasons for non-participation at each stage	Figure 1
		(c) Consider use of a flow diagram	Figure 1
Descriptive data	14*	(a) Give characteristics of study participants (eg demographic, clinical, social) and information on exposures and potential confounders	Table 2
		(b) Indicate number of participants with missing data for each variable of interest	Figure 1
		(c) Summarise follow-up time (eg, average and total amount)	

For peer review only

For peer review only - http://bmjopen.bmj.com/site/about/guidelines.xhtml

Outcome data	15*	Report numbers of outcome events or summary measures over time	7 and Table 2
Main results	16	(*a*) Give unadjusted estimates and, if applicable, confounder-adjusted estimates and their precision (eg, 95% confidence interval). Make clear which confounders were adjusted for and why they were included	Table 2
		(*b*) Report category boundaries when continuous variables were categorized	
		(*c*) If relevant, consider translating estimates of relative risk into absolute risk for a meaningful time period	
Other analyses	17	Report other analyses done—eg analyses of subgroups and interactions, and sensitivity analyses	
**Discussion**			
Key results	18	Summarise key results with reference to study objectives	12
Limitations	19	Discuss limitations of the study, taking into account sources of potential bias or imprecision. Discuss both direction and magnitude of any potential bias	14-15
Interpretation	20	Give a cautious overall interpretation of results considering objectives, limitations, multiplicity of analyses, results from similar studies, and other relevant evidence	12-13
Generalisability	21	Discuss the generalisability (external validity) of the study results	12
**Other information**			
Funding	22	Give the source of funding and the role of the funders for the present study and, if applicable, for the original study on which the present article is based	17

*Give information separately for exposed and unexposed groups.

Note: An Explanation and Elaboration article discusses each checklist item and gives methodological background and published examples of transparent reporting. The STROBE checklist is best used in conjunction with this article (freely available on the Web sites of PLoS Medicine at http://www.plosmedicine.org/, Annals of Internal Medicine at http://www.annals.org/, and Epidemiology at http://www.epidem.com/). Information on the STROBE Initiative is available at http://www.strobe-statement.org.

For peer review only
